# A systematic review of the overlap of fluid biomarkers in delirium and advanced cancer-related syndromes

**DOI:** 10.1186/s12888-020-02584-2

**Published:** 2020-04-22

**Authors:** Ingrid Amgarth-Duff, Annmarie Hosie, Gideon Caplan, Meera Agar

**Affiliations:** 1 University of Technology Sydney, Faculty of Health, IMPACCT -Improving Palliative, Aged and Chronic Care through Clinical Research and Translation, Sydney, NSW Australia; 2 Prince of Wales Clinical School, University of New South Wales, Sydney, NSW Australia; 3 Department of Geriatric Medicine, Prince of Wales Hospital, Sydney, NSW Australia; 4 South West Sydney Clinical School, University of New South Wales, Liverpool, New South Wales Australia; 5 Clinical Trials, Ingham Institute of Applied Medical Research, Liverpool, New South Wales Australia

**Keywords:** Delirium, Biomarker, Advanced cancer, Review

## Abstract

**Background:**

Delirium is a serious and distressing neurocognitive disorder of physiological aetiology that is common in advanced cancer. Understanding of delirium pathophysiology is largely hypothetical, with some evidence for involvement of inflammatory systems, neurotransmitter alterations and glucose metabolism. To date, there has been limited empirical consideration of the distinction between delirium pathophysiology and that of the underlying disease, for example, cancer where these mechanisms are also common in advanced cancer syndromes such as pain and fatigue. This systematic review explores biomarker overlap in delirium, specific advanced cancer-related syndromes and prediction of cancer prognosis.

**Methods:**

A systematic review (PROSPERO CRD42017068662) was conducted, using MEDLINE, PubMed, Embase, CINAHL, CENTRAL and Web of Science, to identify body fluid biomarkers in delirium, cancer prognosis and advanced cancer-related syndromes of interest. Studies were excluded if they reported delirium tremens only; did not measure delirium using a validated tool; the sample had less than 75% of participants with advanced cancer; measured tissue, genetic or animal biomarkers, or were conducted post-mortem. Articles were screened for inclusion independently by two authors, and data extraction and an in-depth quality assessment conducted by one author, and checked by two others.

**Results:**

The 151 included studies were conducted in diverse settings in 32 countries between 1985 and 2017, involving 28130 participants with a mean age of 69.3 years. Seventy-one studies investigated delirium biomarkers, and 80 studies investigated biomarkers of an advanced cancer-related syndrome or cancer prognosis. Overall, 41 biomarkers were studied in relation to both delirium and either an advanced cancer-related syndrome or prognosis; and of these, 24 biomarkers were positively associated with either delirium or advanced cancer syndromes/prognosis in at least one study. The quality assessment showed large inconsistency in reporting.

**Conclusion:**

There is considerable overlap in the biomarkers in delirium and advanced cancer-related syndromes. Improving the design of delirium biomarker studies and considering appropriate comparator/controls will help to better understanding the discrete pathophysiology of delirium in the context of co-existing illness.

## Background

Delirium is a very common cause of acute cognitive change in people with advanced cancer [1] and is associated with increased morbidity and mortality [2, 3]. Delirium is a serious and complex neurocognitive disorder characterized by acute deterioration in attention, awareness and cognition, variously affecting memory, language and visuospatial ability, orientation and perception [4].

Delirium occurs in people who are medically unwell, due to the underlying disease which has put them at risk (e.g. dementia, cancer, infection, renal impairment) or intercurrent problems, and the subsequent medical treatment (e.g. surgery, medication) . Delirium can occur for any person, with those who are older, have advanced illness, and/or prior cognitive impairment most at risk [5]. The prevalence of delirium in patients with advanced cancer in oncology and palliative care settings is higher than that in most other settings, including geriatrics [1, 6–9]. A systematic review of palliative care patients (with 98.9% of participants with advanced cancer), reported delirium incidence rates between 3% and 45%. Delirium prevalence ranged from 13.3% to 42.3% at admission to hospital, and 25% to 62% during admission. Delirium prevalence increased up to 88% in the hours to days before death [1].

The pathophysiology of delirium is poorly understood, and largely hypothetical. Current hypotheses include: neuronal ageing, neuroinflammation, oxidative stress, neuroendocrine dysregulation, disruption to the circadian rhythm, and neurotransmitter dysregulation [10, 11]. A reduction in glucose metabolism seen in people with delirium is a model with developing evidence [12, 13]. Collectively, the biological correlates of delirium are referred to as ‘delirium biomarkers’. A biomarker is a biological molecule found in blood, other body fluids, or tissues that is a sign of a normal or abnormal process, or of a condition or disease [14]. Biomarkers are most commonly studied to investigate their correlation with a disease in order to better understand its underlying pathophysiology, and subsequently inform prevention and treatment strategies for that disease. A challenge for the field of delirium research is that correlation may exist between biomarkers of delirium and those of the patient’s disease or injury which placed them at increased risk of delirium, or which precipitated it (for example sepsis or hip fracture). Such correlation should be factored into delirium biomarker research, yet rarely has been. Better understanding of the interplay between delirium pathophysiology and that of correlated conditions and diseases, for example, cancer (the focus of this review), is crucial to develop more effective prevention and treatment of delirium.

We therefore conducted a systematic review of the literature to explore the overlap between biomarkers that have been studied in delirium and biomarkers that have been studied in cancer-related syndromes. Our aim was to identify biomarkers associated with delirium and with specific clinical situations in advanced cancer (namely prognosis; cognitive impairment, anorexia cachexia, cancer pain, cancer-related fatigue, and sickness behavior); and to evaluate the nature and extent of overlap of the findings.

## Methods

A systematic review according to the Preferred Reporting Items for Systematic Reviews and Meta-Analyses (PRISMA) [15] was conducted. In July 2017, two separate searches were conducted in MEDLINE, PubMed, Embase, CINAHL, CENTRAL, and Web of Science. The first was for literature of delirium biomarkers; the second was for literature of biomarkers in advanced cancer-related syndromes. Primary terms for the delirium search were: ‘delirium’ and ‘biomarker’. Search terms for the cancer search were: ‘cancer’, ‘neoplasms’, ‘metastasis’, ‘fatigue’, ‘sickness behavior’, ‘cancer pain’, ‘cachexia’, and ‘prognosis’. Additional terms which encompassed commonly researched biomarkers were also included. Filters in Medline were: 1: Humans; 2. English language and 3. Published from 1980 onward (when delirium was first included in the *DSM, Third Edition (DSM-III)*)*.* Search terms and filters were tailored to each subsequent database, as required. The full search strategy is provided in Additional file 1. Reference lists of included studies and relevant systematic reviews and meta-analyses identified in the search were examined for additional eligible studies.

We included English language studies published in peer-reviewed journals that reported body fluid biomarkers in adult participants with delirium, cancer prognosis or an advanced cancer-related syndrome of interest. Studies were excluded if they reported delirium tremens only; did not measure delirium using a validated tool; the sample had less than 75% of participants with advanced cancer; measured tissue, genetic or animal biomarkers, or were conducted post-mortem. Protocols and ongoing studies were also excluded. Based on the expert knowledge of the authors in both delirium and cancer, the advanced cancer-related syndromes and prognosis were chosen based on the potential biological plausibility that the pathophysiological mechanisms could overlap with that of delirium. We limited the search to advanced cancer as this is the cancer population with the highest prevalence of both delirium and the cancer-related syndromes of interest.

The following definitions were used in this review:

- ***Anorexia cachexia:*** A complex metabolic syndrome of involuntary weight loss associated with cancer and some other palliative conditions [16].
- ***Cancer related fatigue:*** A distressing, persistent, subjective sense of physical, emotional, and/or cognitive tiredness or exhaustion related to cancer and/or cancer treatment that is not proportional to recent activity and interferes with usual functioning [17].
- ***Cancer-related pain***: An unpleasant sensory and emotional experience associated with actual or potential tissue damage, or described in terms of such damage [18].
- ***Cancer-related cognitive impairment:*** Cognitive impairment that is commonly experienced by cancer patients and those in remission. The cognitive domains most commonly affected are memory, concentration, information processing speed and executive function [19].
- ***Sickness behaviour:*** The coordinated set of behavioural changes that develop in sick individuals during the course of an infection. Sickness behavior is also seen in other illness including cancer [20, 21].
- ***Cancer prognosis:*** The likely outcome or course of the disease; the chance of recovery or recurrence. Cancer prognosis is assessed by cancer-specific survival, overall survival, progression free survival or relative survival [22].

Search results were imported into Endnote X7 software, duplicates removed and then exported into Covidence^TM^ (www.covidence.org). Two reviewers per search (IAD and AH: delirium search, IAD and MA: cancer search) independently applied eligibility criteria for both searches and examined title and abstracts. Exclusions were documented only for articles that required full-text to make a formal decision. Inter-reviewer disagreement on included studies was discussed to resolve any discrepancies, with the third reviewer consulted when required. Data extraction was conducted by one reviewer (IAD) using Excel (2016) with two other reviewers (MA and AH) providing input and oversight. Data extraction was guided by the REporting recommendations for tumor MARKer prognostic studies (REMARK) checklist [23].

In the absence of a gold standard risk of bias assessment for biomarker studies, one reviewer (IAD) applied an adaptation of the REMARK checklist [23] to assess the methodological quality of the included studies, with 10% verification by two other reviewers (MA and AH).

The heterogeneity of data precluded performing a meta-analysis; we therefore reported the data using a narrative synthesis approach with text and tabular summaries. The synthesis was structured according to the overlap of the biomarkers in delirium, cancer prognosis and the cancer syndromes, the biomarker type, assay used, and numbers and proportions of participants who had delirium and advanced cancer. We defined ‘overlap’ as any biomarker that was studied in both a delirium study and an advanced cancer syndrome study.

## Results

**Fig. 1:**
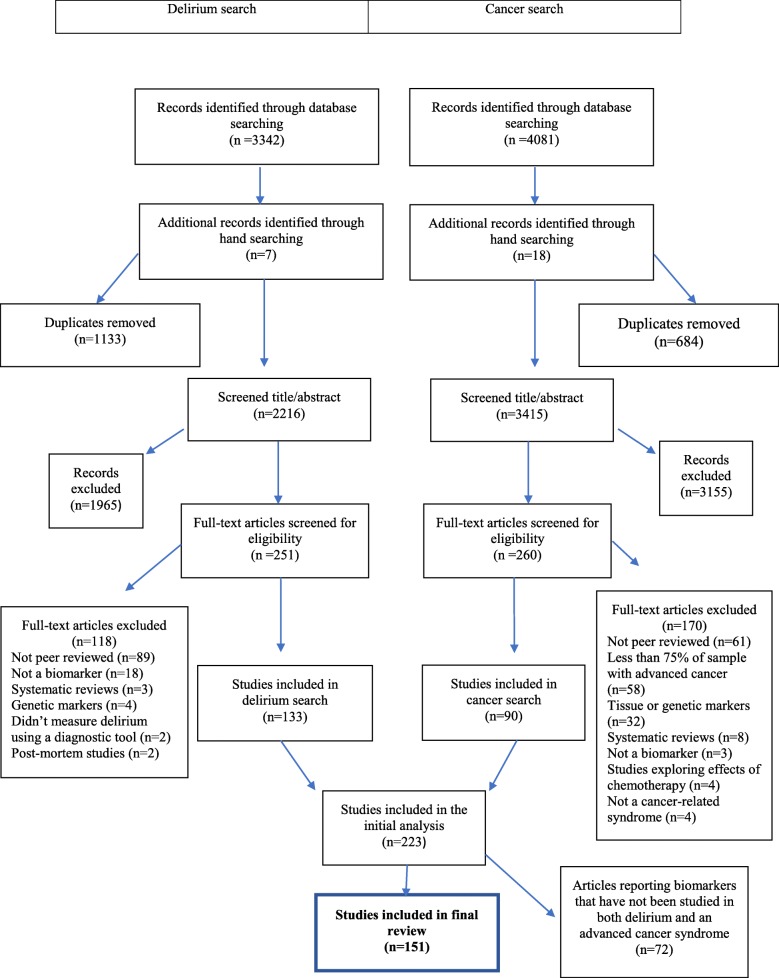
PRISMA flow diagram of search results

The delirium search yielded 3342 articles and the cancer syndromes search 4081, giving a total of 7423 articles. An additional 25 articles were found through the hand search. After removal of 1817 duplicates and 5120 articles through title and abstract screening, we reviewed 511 full text papers and subsequently excluded 288. After initial analysis, a further 72 were excluded as they did not report a biomarker studied in delirium and advanced cancer. This resulted in a total of 151 articles included in this review: 71 reported biomarkers studied in delirium, and 80 reported biomarkers studied in a cancer syndrome or prognosis (Figure 1).

The 151 studies were conducted between 1985 and 2017 in Europe (*n*=86), Asia (*n*=33), The Americas (*n*=27), Australia (*n*=2), and multiple regions (*n*=3). Studies were set in a large range of settings, with the most common in general hospital settings (*n*=111; 73%). Thirty-nine studies (26%) did not report the setting. Sample sizes ranged from 7-2456, with relatively even numbers of male and female participants (55.4% male). Ninety nine articles reported a mean age, with an overall weighted mean age of 69.3 years. Of the 37 articles that reported the median age of participants, the overall median age was 54.5 years. The overall age of participants in the remaining 15 articles was not possible to determine (Additional files 2 and 3). Blood biomarkers were examined in 138 studies, 4 studies examined biomarkers in cerebrospinal fluid (CSF), 3 in urine, and 16 (11%) did not report the type of biological material. Of the studies that reported the assay technique, diverse assays were used (n=20), with Enzyme-linked immunosorbent assay (ELISA) being the most common (n=62; 58%). Forty-four studies (29%) did not report the specific assay used. Of these, 21 studies (48%) were routinely measured biomarkers **(**Tables 1 and 2).

**Table 1:** Characteristics of assays and main findings of included delirium studies*

Author and year	Participants				Endpoints	Biomarkers studied	Biological material	Assay method	Covariates accounted for in multivariate analysis	Results	
	Total (N)	Sample	Total participants with cancer/total participants in the study	Number of delirium with cancer/total number delirium (%)						Positive association with at least one delirium endpoint **	Negative association
Egberts *et al.* (2017) [24]	86	Aged ≥65 admitted to geriatrics	Not measured/NR	Not measured/NR	Delirium presence	CRP, NLR	Blood	Flow cytometry	Age, gender, the CCI score, CRP level, and WBC counts	NLR	CRP
Kozak *et al.* (2017) [25]	60	Patients with acute ischemic stroke	Not measured/NR	Not measured/NR	Delirium presence	TNF-α, IL-1β, IL-18, BDNF, NSE	Serum	ELISA	No multivariate analysis	None	TNF-α, IL-1β, IL-18, BDNF, NSE
Tomasi *et al.* (2017) [26]	38	Patients with sepsis-associated delirium and non-sepsis associated delirium^a^	Not measured/NR Not measured/NR	Not measured/NR	Delirium presence	IL-6, IL-8, IL-10, BDNF, VCAM-1, ICAM-1, MPO, cathepsin, PDGF-AA, PDGF-AB/BB, RANTES, PAI, NCAM	Plasma	ELISA	No multivariate analysis	IL-6, IL-10, RANTES, VCAM-1, ICAM-1, PDGF-AB/BB	IL-8, MPO, BDNF, NCAM, PDGF-AA, PAI, Cathepsin D
Vasunilashorn *et al.* (2017) [27]	560	Patients ≥70 undergoing major non-cardiac surgery^a^	Not measured/NR	Not measured/NR	-Delirium incidence -Delirium duration -Delirium severity	CRP	Plasma	ELISA	Age, sex, surgical procedure, anesthesia route, CCI and POST-OP infectious complications	CRP	None
Chu *et al.* (2016) [28]	103	Patients aged ≥70 admitted for acute or elective vertebral, knee, or hip surgery	Not measured/NR	Not measured/NR	Delirium incidence	IGF-1	Serum	ELISA	MMSE and age	None	IGF-1
Dillon *et al.* (2016) [28]	Entire sample (n-566); pooled sample (n=150)	Dementia-free adults ≥70 years old undergoing major scheduled non-cardiac surgery^a^	Advanced cancer excluded; other cancer stages NR	Advanced cancer excluded; other cancer stages NR	Delirium incidence	Proteomics^b^	Plasma	ELISA	No multivariate analysis	CRP (PRE-OP, PACU, POD2)	CRP (PO1MO)
Guo *et al.* (2016) [29]	572	Aged ≥65 with hip fractures undergoing THA^a^	Not measured/NR	Not measured/NR	-Delirium presence -Delirium prevalence	CRP, Alb, Hb	Blood	NR	NR	CRP, Alb, Hb	None
Karlicic *et al.* (2016) [30]	120	Patients with delirium in the psychiatric ICU	None	Cancer excluded	Lethal outcome	CRP	NR	NR	Age, pneumonia and CRP	CRP	None
Neerland *et al.* (2016) [31]	149	Patients with acute hip fracture	Advanced cancer excluded, other cancer stages NR	Advanced cancer excluded, other cancer stages NR	Delirium presence	CRP, IL-6, sIL-6R	CSF	ELISA	No multivariate analysis	CRP^b^	sIL-6R, IL-6
**Shen*et al.*(2016)** [32]	140	Patients ≥65 undergoing elective gastrointestinal tumor resection^a^	140/140 (100)	36/36 (100)	-Delirium incidence -Delirium severity	IGF-1, CRP, IL-6	Serum	ELISA	NR	IGF-1, CRP, IL-6	None
**Sun*et al.*(2016)** [33]	112	Oral cancer patients^a^	112/112 (100)	56/56 (100)	Delirium incidence	IL-6, CRP, PCT, cortisol, AB1-40	Blood	ELISA	No multivariate analysis	IL-6, CRP, PCT, cortisol, AB1-40	None
Yen *et al.* (2016) [34]	98	Patients undergoing elective knee replacement surgery	Not measured/NR	Not measured/NR	Delirium incidence	IGF-1	Serum	ELISA	Obstructive sleep apnea, IGF-1 and diabetes	None	IGF-1
**Avila-Funes*et al.*(2015)** [35]	141	Patients aged ≥70 admitted to tertiary care hospital	37/141 (26.2)	6/23 (26)	Delirium incidence	Cortisol, E2	Blood	Radioimmunoassay	Age, BMI, comorbidity, MMSE, previous history of delirium, BUN/Cr ratio, and cortisol levels	E2	Cortisol
**Brum*et al.*(2015)** [36]	70	Oncology inpatients^a^	45-70 (64.2)	17/17 (100)	Delirium presence	BDNF, TNF-α	Serum	ELISA + Flow cytometry	No multivariate analysis	None	BDNF, TNF-α
Egberts *et al.* (2015) [37]	86	Patients admitted to Internal Medicine and Geriatrics^a^	Not measured/NR	Not measured/NR	Delirium presence	NP, IL-6, IGF-1	Plasma	HPLC	Age, gender and the CCI, and those including NP were adjusted for age, gender, CCI, tertiles of eGFR and CRP	NP, IL-6, IGF-1	None
**Foroughan*et al****.* (2015) [38]	200	Elderly patients admitted to general hospital	18/200 (9)	12/44 (27)	Delirium presence	CRP, Hb	Blood	NR	NR	CRP, Hb	None
Skrede *et al.* (2015) [39]	10	Patients with hip fracture	Not measured/NR	Not measured/NR	Delirium incidence	MCP-1	Serum	ELISA	No multivariate analysis	MCP-1	None
Vasunilashorn *et al.* (2015) [40]	566	Patients ≥70 undergoing major non-cardiac surgery^a^	Not measured/NR	Not measured/NR	Delirium incidence	IL-1Β, IL-2, IL-4, IL-5, IL-6, IL-8, IL-10, IL-12, IFN-γ, GM-CSF, TNF-α, VEGF	Plasma	Luminex assay	No multivariate analysis	IL-1Β, IL-2, IL-6, IL-8, IL-12, VEGF, IL-5, TNF-α	GM-CSF, IFN-γ, IL-10, IL-4
Alexander *et al.* (2014) [41]	77	ICU patients requiring mechanical ventilation	Not measured/NR	Not measured/NR	-Delirium presence -Delirium duration	IL-6, IL-8, IL-10, APOE	Serum	ELISA	Age, sex, APACHE III, CCI, 24-hour propofol dose, 24-hour narcotic dose, and 24-hour benzodiazepine dose.	APOE	IL-10, IL-8, IL-6
Baranyi *et al.* (2014) [42]	34	Patients undergoing surgery for CPB^a^	Not measured/NR	Not measured/NR	Delirium incidence	sIL-2R	Serum	ELISA	No multivariate analysis	sIL-2R	None
Cape *et al.* (2014) [43]	43	Patients >60 years old with hip fracture	Not measured/NR	Not measured/NR	-Delirium incidence -Delirium prevalence	IL-1β, IFN-γ, GFAP, IGF-1, IL-1RA	CSF	ELISA	Presence of prior dementia	IL-1β, IL-1RA^c^	GFAP, IFN-γ, IGF-1
Capri *et al.* (2014) [44]	351	Patients admitted for any kind of emergency or elective surgery^a^	Comorbidity measured, cancer NR	Comorbidity measured, cancer NR	Delirium presence	IL-1β, IL-2, IL-6, IL-8, IL-10, TNF-α	Plasma	ELISA	Age, comorbidity, ADL, IADL, HADS and pre-op benzodiazepines intake	IL-6, IL-2	IL-8, IL-10, IL-1β (UDL), TNF-α (UDL)
Chen *et al.* (2014) [45]	372	Patients aged ≥65 who underwent surgery for a femoral neck fracture or an intertrochanteric fracture^a^	Not measured/NR	Not measured/NR	Delirium presence	LP	Plasma	ELISA	Age, ASA, type of surgery and plasma leptin level	LP	None
Hatta *et al.* (2014) [46]	29	Patients aged 65-89 admitted to hospital due to an emergency	Not measured/NR	Not measured/NR	Delirium incidence	NK cell activity, IL-1β	Blood	ELISA	No multivariate analysis	NK cell activity	IL-1β
Kazmierski *et al.* (2014) [47]	113	ICU patients scheduled for CABG surgery with CPB	Not measured/NR	Not measured/NR	Delirium incidence	Cortisol, IL-2, TNF-α, HCY, cobalamin	Serum	CLIA	NR	Cortisol, IL-2, TNF-α, HCY	Cobalamin
Ritchie *et al.* (2014) [48]	710	Patients admitted to a Medical Acute Admission Unit	Not measured/NR	Not measured/NR	-Delirium incidence -Delirium severity	CRP	NR	NR	NR	CRP	None
Ritter *et al.* (2014) [49]	78	ICU patients	Not measured/NR	Not measured/NR	Delirium presence	TNF-α, STNFR-1, STNFR2, APN, IL-1β, IL-6, IL-10	Plasma	ELISA	Sedation and sepsis	STNFR-1, STNFR2, IL-1β	TNF-α, IL-6, IL-10
Zhang *et al.* (2014) [50]	223	ICU patients	Not measured/NR	Not measured/NR	Delirium presence	CRP	Plasma	i-CHROMATM	Age, sex, APACHE II, intubation status, living alone, physical restraint, alcohol drinking, smoking, type of medical condition, and hospital LOS before ICU admission	CRP	None
Cerejeira *et al.* (2013) [51]	101	Patients ≥60 years without dementia undergoing elective hip arthroplasty^a^	Not measured/NR	Not measured/NR	Delirium incidence	Cortisol, IGF-1, CRP, IL-6, IL-8, IL-10	Plasma	ELISA	No multivariate analysis	Cortisol	CRP, IL-6, IL-8, IL-10, IGF-1
Colkesen *et al.* (2013) [52]	52	Patients with ACS admitted to coronary ICU^a^	Not measured/NR	Not measured/NR	Delirium presence	Cortisol, troponin I, MB-CK	Serum	CLIA	NR	Cortisol	Troponin I, MB-CK
Kazmierski *et al.* (2013) a [53]	113	ICU patients scheduled for CABG surgery with CPB	Not measured/NR	Not measured/NR	Delirium incidence	Cortisol, IL-2	Plasma	CLIA	NR	Cortisol^d^, IL-2	None
Kazmierski *et al.* (2013) b [54]	113	ICU patients scheduled for CABG surgery with CPB	Not measured/NR	Not measured/NR	Delirium incidence	IL-2, TNF-α	Plasma	CLIA	NR	Il-2, TNF-α	None
Liu *et al.* (2013) [55]	338	Patients aged ≥60 undergoing major non-cardiac surgery^a^	Not measured/NR	Not measured/NR	Delirium incidence	IL-6	Blood	ELISA	Age, education, history of coronary artery disease, alcoholism, PRE-OP ASA ≥ 3, PRE-OP NYHA ≥ 2, PRE-OP MMSE score ≤ 24, PRE-OP serum IL-6 ≥ 7.5 ph/ml, POST-OP serum IL-6, POST-OP VAS pain level	IL-6	None
Plaschke *et al.* (2013) [56]	114	1. Patients following heart surgery^a^ 2. Patients on the non-cardiac ICU^a^	Not measured/NR	Not measured/NR	Delirium incidence	IL-6	Plasma	ELISA	No multivariate analysis	None	IL-6
Skrobik *et al.* (2013) [57]	99	ICU patients^a^	Not measured/NR	Not measured/NR	Drug-induced coma and delirium	TNF-α, IL-1β, IL-1RA, IL-6, IL-8, IL-10, IL-17, MIP-1B, MCP-1	Blood	BCA	Fentanyl, midazolam, CYP3A4/5, P-gp inhibitors	IL-6	TNF-α, IL-17, IL-8, MCP-1, IL-1RA, MIP-1B, IL-10, IL-1β
Westhoff *et al.* (2013) [58]	61	Patients ≥75 admitted for surgical repair of acute hip fracture^a^	Not measured/NR	Not measured/NR	Delirium incidence	EGF, eotaxin, FGF-2, Flt-3L, Fractalkine, G-CSF, GM- CSF, IFN-a2, IFN-γ, IL-1RA, IL-1α, IL-1β, IL-2, sIL-2Ra, IL-3, IL-4, IL-5, IL-6, IL-7, IL-8, IL-9, IL-10, IL-12p40, IL-12p70, IL-13, IL-15, IL-17, IP-10, MCP-1, MCP-3, MDC, MIP-1α, MIP-1β, PDGF-AA, PDGF-AB/BB, RANTES, sCD40L, TGF-α, TNF-α, TNF-β, VEGF	Blood + CSF	Lumbar punctures and Luminex assays	No multivariate analysis	Flt-3L, IL-1RA, IL-6	EGF, eotaxin, FGF-2, Fractalkine, G-CSF, GM- CSF, IFN-a2, IFN-γ, IL-1α, IL-1β, IL-2, sIL-2Ra, IL-3, IL-4, IL-5, IL-7, IL-8, IL-9, IL-10, IL-12p40, IL-12p70, IL-13, IL-15, IL-17, IP-10, MCP-1, MCP-3, MDC, MIP-1α, MIP-1β, PDGF-AA, PDGF-AB/BB, RANTES, sCD40L, TGF-α, TNF-α, TNF-β, VEGF
Bakker *et al.* (2012) [59]	201	Patients undergoing cardiac surgery	Not measured/NR	Not measured/NR	Delirium incidence	Cre	Plasma	NR	NR	Cre	None
Baranyi *et al.* (2012) [60]	34	Patients undergoing surgery for cardiopulmonary bypass^a^	Not measured/NR	Not measured/NR	Delirium incidence	Alb, CRP	Serum	NR	No multivariate analysis	Alb	CRP
Cerejeira *et al.* (2012) [61]	101	Patients aged ≥60 undergoing elective total hip arthroplasty^a^	Not measured/NR	Not measured/NR	Delirium incidence	IL-8, IL-1β, IL-6, IL-10, TNF-α, CRP, AChE, BuChE	Blood	ELISA (Multiplex assay)	No multivariate analysis	AChE, BuCHE	CRP, IL-1β, TNF-α, IL-6, IL-10
Girard *et al.* (2012) [62]	138	Mechanically ventilated ICU patients^a^	Not measured/NR	Not measured/NR	Delirium incidence	CRP, MMP-9, MPO, NGAL, sTNFR1, D-dimer, protein C, PAI-1, VWF	Plasma	ELISA	Age, severity of illness, and severe sepsis	MMP-9, Protein C, sTNF-R1	CRP, MPO, NGAL, D-dimer, PAI-1, VWF
Osse *et al.* (2012) [63]	125	Patients ≥70 undergoing elective cardiac surgery	Not measured/NR	Not measured/NR	Delirium incidence	NP, BH4, HVA, Glu, Ser, Gly, Cit, Tau, Arg, Met, Try, Tyr, Phe, Leu, Ile, Val, Try:LNAA, Tyr:LNAA, Phe:LNAA, Phe:tyr, Cit:arg, Tau:Ser 9 met	Plasma	HPLC	BH4, total biopterin, HVA, ratios of Trp:LNAA, tyr: LNAA, phe: LNAA, phe: Tyr, Cit:Arg, TSM ratio; baseline CRP, plasma urea, cre, age, sex, type of surgery, acute cardiac surgical risk factors, EuroSCORE, MMSE, pre-op anxiety and depression, and chronic medical comorbidity	NP, HVA	BH4, Glu, Ser, Gly, Cit, Tau, Arg, Met, Try, Tyr, Phe, Leu, Ile, Val, Try:LNAA, Tyr:LNAA, Phe:LNAA, Phe:tyr, Cit:arg, Tau:Ser 9 met
Bisschop *et al.* (2011) [64]	143	Patients undergoing surgery for hip fracture	Not measured/NR	Not measured/NR	-Delirium presence -Delirium severity	Cortisol, insulin, glucose	Blood	NR	Sex, age, pre-existing cognitive impairment, pre-existing functional impairment, cortisol, glucose, insulin, insulin:glucose	Cortisol	Glucose, insulin
Holmes *et al.* (2011) [65]	222	Patients with mild to severe AD	Not measured/NR	Not measured/NR	-Presence of sickness behaviour -Delirium incidence	IL-6, TNF-α, CRP	Blood	ELISA	Baseline ADAS score, age, gender, and the presence of delirium	None	Il-6, TNF-α, CRP
Lee *et al.* (2011) [66]	65	Patients ≥65 who had undergone hip surgery^a^	Not measured/NR	Not measured/NR	Delirium incidence	CRP	Blood	NR	No multivariate analysis	None	CRP
McGrane *et al.* (2011) [67]	87	Mechanically ventilated, medical and surgical ICU patients^a^	Not measured/NR	Not measured/NR	Delirium/coma-free days	PCT, CRP	Blood	TRACE Assay analysis	Age, APACHE II, sedation group (dexmedetomidine vs. lorazepam), and sepsis	PCT	CRP
Morandi *et al.* (2011) [68]	110^e^	Mechanically ventilated medical ICU patients	Not measured/NR	NR	Delirium presence	IGF-1	Blood	Radioimmunoassay	Age, severe sepsis and APACHE II		IGF-1
Van der Boogaard *et al.* (2011) a [69]	100	ICU patients^a^	Not measured/NR	Not measured/NR	Delirium presence	TNF-α, IL-1β, IL-6, IL-8, IL-17, IL-18, MIF, IL-1RA, IL-10, MCP-1, HNP-1, CRP, PCT, Ab1-42, Ab1-40, S100B, cortisol	Plasma	Luminex assay, immunologic detection, and an immunometric assay	NR	**Delirium vs non-delirium:** IL-8^f^, IL-10^g^, Ratio A*β*_1-42/40_, TNF-α, IL-6, MIF, IL-1RA, MCP-1, PCT, cortisol, ABN-42 **Inflamed delirium vs non-inflamed delirium:** IL-8, TNF-α, IL-18, IL-1RA, MCP-1, PCT, CRP, ratio A*β*_1-40/N-40,_ ratio A*β*_N-42/40,_	**Delirium vs non-delirium:** IL-1Β, IL-17, IL-18, HNP, CRP, S100B, Tau, Ratio Tau/A*β*_1-42,_ A*β*_1-42,_ A*β*_1-40,_ A*β*_N-42,_ A*β*_N-40,_ Ratio A*β*_N-42/40,_ Ratio A*β*_1-42/N-42,_ Ratio A*β*_1-40/N-40_ **Inflamed delirium vs non-inflamed delirium:** IL-1β, IL-6, MIF, IL-10, cortisol, ABN-42, IL-1Β, IL-17, HNP, S100B, Tau, tau/AB1-42, Ratio Tau/A*β*_1-42,_ A*β*_1-42,_ Ratio A*β*_1-42/N-42_A*β*_1-40,_ Ratio A*β*_1-42/40,_ A*β*_N-42,_ A*β*_N-40_
Van der Boogaard *et al.* (2011) b [70]	20	ICU patients	Not measured/NR	Not measured/NR	Delirium presence	Proteomics^h^	Urine + Blood	NR	No multivariate analysis		CRP, Cre
Burkhart *et al.* (2010) [71]	113	Patients aged ≥65 undergoing elective cardiac surgery with CPB	Not measured/NR	Not measured/NR	Delirium presence	CRP	NR	NR	EuroSCORE, Leucocytes, CRP max, Fentanyl intraoperatively, duration of mechanical ventilation, packed RBC, and treated PONV	CRP	None
Mu *et al.* (2010) [72]	243	Patients undergoing elective CABG surgery	Not measured/NR	Not measured/NR	Delirium incidence	Cortisol	Serum	CLIA	Age, history of diabetes mellitus, PRE-OP LVEF, PRE-OP NYHA, preop EuroSCORE score, duration of surgery, POST-OP APACHE II, serum cortisol, POST-OP LVEF, POST-OP complications (within 1 day)	Cortisol	None
Pearson *et al.* (2010) [73]	20	Patients ≥60 with acute hip fracture awaiting surgery^a^	Not measured/NR	Not measured/NR	Delirium presence	Cortisol	CSF + serum	ELISA	No multivariate analysis	Cortisol	None
Plaschke *et al.* (2010) [74]	114^i^	Patients undergoing elective CABG^a^	Not measured/NR	Not measured/NR	Delirium incidence	Cortisol, IL-6	Plasma	ELISA	No multivariate analysis	IL-6, cortisol	None
Tsruta *et al.* (2010) [75]	103	ICU patients^a^	Not measured/NR	Not measured/NR	-Delirium incidence -Delirium prevalence	CRP	Serum	Immunoturbidimetry	Age, APACHE II, coexistence of infection, use of a mechanical ventilator and length of ICU stay	CRP	None
Van Munster *et al.* (2010) [76]	120	Patients ≥65 admitted for hip fracture surgery	Not measured/NR	Not measured/NR	Delirium presence	Cortisol, IL-6, IL-8, S100B	Plasma	CBA	Age, infection, pre-existent cognitive and functional impairment	Cortisol, IL-6, IL-8, S100B	None
Adamis *et al.* (2009) [77]	67	Patients aged ≥70 admitted to elderly care unit	Not measured/NR	Not measured/NR	-Delirium incidence -Delirium severity	APOE, IL-1α, IL-1β, IL-1RA, IL-6, TNF-α, IGF-1, IFN-γ, LIF	Serum	ELISA	No Multivariate analysis	IGF-1, IFN-γ, IL-1RA,	APOE, IL-1α, IL-1β, IL-6, TNF-α, LIF
Van Munster *et al.* (2009) [78]	120	Patients ≥65 admitted for hip fracture surgery	Not measured/NR	Not measured/NR	Delirium incidence	S100B, NSE	Blood	ECLIA	No multivariate analysis	S100B	NSE
Lemstra *et al.* (2008) [79]	68	Patients undergoing surgery for hip fracture	Not measured/NR	Not measured/NR	Delirium incidence	CRP, IL-6, IGF-1	Blood	ELISA	No multivariate analysis	None	CRP, IL-6, IGF-1
Pfister *et al.* (2008) [80]	16^j^	Patients with sepsis	Not measured/NR	Not measured/NR	Sepsis-related delirium presence	CRP, IL-6, S-100B, cortisol	Serum	Solid-phase enzyme-labelled chemiluminescent sequential immunometric assay	No multivariate analysis	CRP, S100B, Cortisol	IL-6
Rudolph *et al.* (2008) [81]	42	Patients undergoing cardiac surgery	Not measured/NR	Not measured/NR	Delirium incidence	IL-1β, IL-1RA, IL-6, IFN-a, TNF-α, TNF-R1, TNF-R2, IL-2, IL-2R, IL-7, IL-12p40_p70, IL-15, IFN-γ, IP-10, IL-4, IL-5, IL-10, IL-13, MIP-1a, MIP-1b, MIG, Eotaxin, RANTES, CCL-2, IL-8, GM-CSF, IL-17, DR5	Serum	ELISA	No multivariate analysis	MIP-1a, MIP-1b, MIG, Eotaxin, RANTES, CCL-2	IL-1β, IL-1RA, IL-6, IFN-a, TNF-α, TNF-R1, TNF-R2, IL-2, IL-2R, IL-7, IL-12p40_p70, IL-15, IFN-γ, IP-10, IL-4, IL-5, IL-10, IL-13, IL-8, GM-CSF, IL-17, DR5
Van Munster *et al.* (2008) [82]	98	Patients ≥65 admitted for hip fracture surgery	Not measured/NR	Not measured/NR	Delirium presence	IL-6, IL-8, IL-12 (TNF-α, IL-1β, and IL-10 excluded from analysis)	Plasma	CBA	No multivariate analysis	Il-6, IL-8	IL-12
Adamis *et al.* (2007) [83]	164	Acutely ill patients admitted to elderly care unit	Not measured/NR	Not measured/NR	-Delirium presence -Delirium resolution	APOE, IL-1α, IL-1β, IL-1RA, IL-6, TNF-α, IGF-1, IFN-γ, LIF, CRP	Serum	ELISA	LogAPACHE II, DRS, CRP, Gender, TNF-α, IFN-g, IGF-I, IL-1RA, and possession of APOE epsilon 4 allele	IGF-1, APOE, IFNγ	IL-6, IL-1α, IL-1β, IL-1RA, TNF-α, LIF, CRP
**de Rooij*et al.*(2007)** [84]	185	Patients aged ≥65 admitted to the Department of Medicine	18/185 (9.7)	9/64 (14)	Delirium presence	IL-1β, IL-6, IL-8, IL-10, TNF-α, CRP	Serum	CBA	Age, cognitive impairment, and infection	IL-6, IL-8	IL-1β, IL-10, TNF-α, CRP
Plaschke *et al.* (2007) [85]	37	ICU patients	Not measured/NR	Not measured/NR	Delirium presence	SAA, IL-6	Blood	ELISA	No multivariate analysis for IL-6	None	SAA, IL-6
White *et al.* (2005) [86]	283	Patients ≥75 from emergency medical admissions	Not measured/NR	Not measured/NR	-Delirium prevalence -Delirium incidence	CRP, Alb, AChE, BuChE, Aspirin esterase, Benzoylcholinesterase	Plasma	ELISA	No multivariate analysis	CRP, Alb, AChE, BuChE, Aspirin esterase, Benzoylcholinesterase	None
Wilson *et al.* (2005) [87]	100	Patients ≥75 suffering from significant physical illness	Not measured/NR	Not measured/NR	Delirium incidence	IGF-1	Plasma	CLIA	Depression, IGF-1 levels and IQCODE scores	IGF-1	None
Beloosesky *et al.* (2004) [88]	32	Patients undergoing surgery for hip fracture	Not measured/NR	Not measured/NR	-Cognition -Post-operative complications (including delirium) -Post-operative function -Mortality	CRP, FBG	Blood	Nephelometric assay	Unclear	CRP	FBG
Robertsson *et al.* (2001) [89]	172	Patients <80 referred to the neuropsychiatric diagnostic unit with suspected dementia	Not measured/NR	Not measured/NR	Delirium presence	Cortisol	Serum	NR	Age, severity of dementia and severity of delirium	Cortisol	None
Van der Mast *et al.* (2000) [90]	296^k^	Patients admitted for elective cardiac surgery	Not measured/NR	Not measured/NR	Delirium incidence	Try, Ile, Val, Met, Leu, Tyr, Phe, Ser, cortisol	Plasma	HPLC	Plasma amino acids; the ratios of Trp/oLNAA, Tyr/oLNAA, and Phe/oLNAA; albumin; cortisol; and thyroid functions.	Trp, Trp:LNAA	Cortisol, Ile, Val, Met, Leu, Tyr, Phe, Ser
Van der Mast *et al.* (1999) [91]	296	Patients admitted for elective cardiac surgery	Not measured/NR	Not measured/NR	Delirium incidence	Alb, cortisol, 5-HT, try, phe, val, leu, Ile, try:tyr:phe	Plasma	HPLC	Age, inclusion as an in-patient, use of nifedipine, MMSE score, GHQ score, DAL score, Albumin, ratio rT3:T3; ratio Phe:oLNAA	Alb, phe: Ile, Phe:Leu, Phe:val, Phe:tyr, Phe:try	Cortisol, 5-HT
Gustafson *et al.* (1993) [92]	155	Stroke patients	Not measured/NR	Not measured/NR	Delirium presence	Cortisol	Plasma	Radioimmunoassay	Intercept, basal plasma cortisol, paresis, age, left-sided brain lesion, sex, anticholinergic medication, post-dexamethasone plasma cortisol	Cortisol	None
McIntosh *et al.* (1985) [93]	7	Male patients admitted to hospital for elective surgery	Not measured/NR	Not measured/NR	Delirium incidence	Cortisol, B-endorphin	Plasma	Radioimmunoassay	No multivariate analysis	Cortisol, B-endorphin	None

* Studies with both delirium and cancer participants are bolded; red coloured biomarkers indicate significance in multivariate analysis

^a^ Dementia was an exclusion criteria

^b^ Only CRP is reported from this study

^c^ Only between incident and prevalent delirium

^d^ Pre-operative and post-operative cortisol remained significantly increased in delirium, however, after controlling for pre-operative depression, only preoperative cortisol concentration remained significant, irrespective of the cortisol level after surgery.

^e^ Only 66 included in the primary analysis

^f^ In inflamed patients only

^g^ In non-inflamed patients only

^h^Only CRP and Cre are reported

^i^ Same cohort as Plaschke et al. 2007

^j^ Only 16 were analysed

^k^ same cohort as Van Der Mast et al. 1999

Abbreviations: *5HIAA* 5-Hydroxyindoleacetic acid, *5-HT* Serotonin, *6-SMT* 6-sulfatoxymelatonin, *8-Iso PGF2a* 8-iso-prostaglandin F2α, *A1A* Alpha-1 antitrypsin, *a-1-AGP* a-1-acid glycoprotein, *AA* Anticholinergic activity, *AB1* Amyloid-B, *AChE* Acetylcholinesterase, *ACS* Acute Coronary Syndromesm, *ADAS* Alzheimer’s Disease Assessment Scale, *ADL* Activities of daily living, *Ala* Alanine, *Alb* Albumin, *AD* Alzheimer’s Disease, *APACHE* Acute Physiology and Chronic Health Evaluation, *APN* Adiponectin, *ANG* Angiopoietin, *APOA1* Apolipoprotein A1, *APOE*: Apolipoprotein E, *Arg* Arginine, *APS* Acute Physiology Score, *ASA* American Society of American Society of Anaesteologists Scale, *BCA* The bicinchoninic acid assay, *BDNF* Brain-Derived Neurotrophic Factor, *BH4* Tetrahydrobiopterin, *BLI* B-Endorphin-Like Immunoreactivity, *BuChE* Butyrylcholinesterase, *C3* Complement C3, *CABG* Coronary Artery Bypass Graft, *CBA* Cytometric bead array immunoassay, *CCI* Charlson Comorbidity Index, *Cit* Citrulline, *CK* Creatine Kinase, *CK-MB* Creatine Kinase-MB, *CLIA* Chemiluminescence immunoassay, *CNTN-1* Contactin-1, *CPB* Cardiopulmonary Bypass, *Cre* Creatinine, *CRP* C-Reactive Protein, *E2* Estrodiol, *FBG* Fibrinogen, *FBLN-1* Fibulin-1, *ECLIA* Electrochemiluminescence immunoassay, *EGF* Epidermal Growth Factor, *FGF-2* Fibroblast Grown Factor, *Flt-3L* FMS-like tyrosine kinase 3 ligand, *GABA* Gamma-Aminobutyric Acid, *G-CSF* Granulocyte Stimulating Factor, *GFAP* Glial Fibrillary Acidic Protein, *GHQ* General Health Questionnaire, *Glu* Glutamic acid, *Gly* Glycine, *GM-CSF* Granulocyte-Macrophage Colony-Stimulating Factor, *HADS* Hospital Anxiety and Depression Scale, *Hb* Haemoglobin, *HCY* Homocysteine, *HNP-1* Defensin, *HP* Haptoglobin, *HPLC* High-performance liquid chromatography, *HVA* Homovanillic Acid, *IADL* Instrumental activities of daily living, *ICU* Intensive care unit, *Ile* Isoleycine, *ICAM-1* Intercellular Adhesion Molecule 1, *IDO* Indoleamine 2, 3-dioxygenase, *IFN* Interferon, *IGF* Insulin- Like Growth Factor, *IL* Interleukin, *IL-1RA* Interleukin-1 Receptor Antagonist, *Ile* Isoleucine, *IP-10* Interferon gamma-induced protein 10, *IQCODE* The Informant Questionnaire on Cognitive Decline in the Elderly, *KYN* Kynurenine, *Leu* Leucine, *LIF* Leukaemia Inhibitory Factor, *LNAA* Large Neutral Amino Acids, *LOS* Length of stay, *LP* Leptin, *Met* Methionine, *MB-CK* MB-isoform of Creatinine Kinase, *MCP* Monocyte Chemotactic Protein, *MDC* Human Macrophage-derived Chemokine, *MIF* Macrophage Migration Inhibitory Factor, *MIG* Monokine induced by Gamma Interferon, *MIP* Macrophage Inflammatory Protein, *MMP-9* Matrix Metalloproteinase- 9, *MMSE* Mini-mental state examination, *MPO* Myeloperoxidase, *MT* Melatonin, *NCAM* Neural Cell Adhesion Molecule, *NGAL* Neutrophil Gelatinase-Associated Lipocalin, *NLR* Neutrophil- Lymphocyte ratio, *NK cells* Natural killer cells, *NP* Neopterin, *NR* Not reported, *NSE* Neuron Specific Enolase, *Orn* Ornithine, *NYHA* New York Heart Association, *PACU* Post-anesthesia care unit, *PAI-1* Plasminogen activator inhibitor-1, *PCT* Procalcitonin, *PDGF* Platelet- Derived Growth Factor, *Phe* Phenylalanine, *pMHPG* Plasma free 3-methoxy-4-hydroxyphenylglycol, *pNF-H* The Phosphorylated Neurofilament H, *PO1MO* 1 month post-operative, *POD2* Post-operative day 2, *PONV* Post-operative nausea and vomiting, *POST-OP* Post-operative, *PRE-OP* Pre-operative, *P-tau* Phosphorylated tau, *RANTES* Chemokine (C-C motif) ligand 5, *RBC* Red blood cell, *S100B* s100 calcium-binding protein B, *sCD40L* Soluble CD40 ligand, *Ser* Serine, *sIL-XR* Soluble IL- X receptor, *SLI* Somatostatin-Like Immunoreactivity, *sTNFR* Soluble Tumor Necrosis Factor Receptor, *Tau* Taurine, *T-tau* Total tau, *TGF-a* Transforming Growth Factor Alpha, *THA* Total Hip Arthroplasty, *TRACE* Time Resolved Amplified Cryptate Emission, *TSH* Thyroid Stimulating Hormone, *TNF* Tumor Necrosis Factor, *Trp* Tryptophan, *TRX* Thioredoxin, *Tyr* Tyrosine, *UDL* Under detection limit, *Val* Valine, *VCAM-1* Vascular Cell Adhesion protein 1, *VEGF* Vascular Endothelial Growth Factor, *vWF* Von Willebrand factor, *ZAG* Zinc-a-2-Glycoprotein

**Table 2:** Characteristics of assays and main findings of included cancer studies*

Author and year	Participants		Endpoints	Biomarkers studied	Biological material	Assay method	Covariates adjusted for in multivariate analysis	Results	
	Total participants (N)	Cases; control						Positive association with at least one endpoint**	Negative association
Amano *et al*. (2017)^a^ [94]	1702	Advanced cancer patients; no control	-Anorexia -Weight loss -Fatigue -Dyspnea -Dysphasia -Edema -Pressure ulcer -ADL disabilities	CRP	NR	NR	Age, gender, primary tumor site, distant metastasis, chemotherapy, ECOG PS, and setting of care	CRP	None
Demiray *et al*. (2017) [95]	87	Participants with advanced cancer; healthy participants without a known chronic disease	-Cachexia -Weight loss -PFS -OS	LP, resistin	Serum	ELISA	NR	LP Multivariate results NR	Resistin* Multivariate results NR
Fogelman *et al*. (2017) [96]	69	Participants with advanced cancer; healthy controls with no cancer diagnosis	Either 10% weight loss or death at 60 days from the start of therapy	APN, bFGF, CXCL-16, FSN, Ghrelin, IGF-1, IL-1β, IL-6, IL-8, Klotho, LP, MCP-4, MK, MSTN, PIF, sTNFR1, sTNFR2, TARC, TNF-α, VEGF, ZAG	NR	NR	Smoking status, best response, pain, difficulty swallowing	MK, IL-1β, CXCL- 16, IL-6, IL-8, TNF-α Multivariate results NR	APN, bFGF, FSN, Ghrelin, IGF-1, Klotho, LP, MCP-4, MSTN, MK, PIF, sTNFR1, sTNFR2, TARC, VEGF, ZAG Multivariate results NR
Luo *et al*. (2017) [97]	217	Participants with advanced cancer; no control	-PFS -OS	FBG, CA-125, NLR, PLR	Serum + Plasma	NR	NR	FBG	CA-125, NLR, PLR
Paulsen *et al*. (2017) [98]	49	Participants with cancer; no control	-Pain -Appetite -Fatigue	CRP, ESR, sTNF-R1, IL-1RA, IL-6, MCP-1, IL-18, MIF, TGF-β1	Serum	ELISA (multiplex assay)	Sex, BMI and age	sTNF-r1, MCP-1, MIF, CRP, IL-6, IL-1RA	IL-18, TGF-β 1, ESR
Amano *et al*. (2016) [99]	1511	Advanced cancer patients; no control	-Survival rate -Mortality rate	CRP	Plasma	Latex-enhanced immunoturbidimetric assay	Age, gender, primary tumor site, distant metastasis, chemotherapy, ECOG PS, and setting of care	CRP	None
Bye *et al*. (2016) [100]	60	Participants with advanced cancer; healthy controls with normal weight	-Cachexia -Survival	IL-10, IFN-γ, LP, APN, TNF-α, IL-6, IGF-1	Serum	ELISA	No multivariate analysis	IL-6	IL-10, IFN-γ, TNF-α, APN, IGF-1
Mitsunga *et al*. (2016) [101]	421	Participants with advanced cancer with low, intermediate and high CRP levels	OS	CRP, NLR	Blood	ELISA (Multiplex assay)	**Retrospective cohort:** Sex, age, ECOG-PS, UICC stage, CA 19-9, prognostic CRP classification; **Prospective cohort**: Sex, age, ECOG-PS, UICC stage, CA 19-9, NLR classification, mGPS, prognostic CRP classification	CRP, NLR	None
Morgado *et al*. (2016) [102]	49	Participants with advanced cancer and fatigue with and without weight loss	-Weight loss -Fatigue	Hb, LDH, Alb, CRP, Cre	Serum + Urine	NR	No multivariate analysis	Alb, CRP	Hb, LDH, Cre
Rodrigues *et al*. (2016) [103]	51	Participants with advanced cancer; no control	Fatigue	IL-1, IL-6, TNF-α, α-1-AGP, GPS (Alb+CRP)	Blood	NR	No multivariate analysis	TNF-α, GPS (Alb+CRP)	None
Srdic *et al*. (2016) [104]	100	Participants with advanced cancer with and without cachexia	-Cachexia -Chemotherapy toxicity -Survival	CRP, IL-6, Alb, Hb	NR	The Bromocresol Purple method	NR	CRP, IL-6, Alb, Hb	None
Wu *et al*. (2016) [105]	55	Participants with advanced cancer; no control	-OS -PFS	NLR, PLR, ALP, LDH	Blood	NR	NR	PLR**,** NLR, LDH	ALP
Bilir *et al*. (2015) [106]	80	Participants with advanced cancer and cachexia; healthy controls with no known chronic disease or weight loss	-OS -Cachexia	Il-1β, IL-1α, IL-6, TNF-α, orexin-A, galanin, TWEAK, TRAF-6, NPY, CRP, Testosterone, Alb, LDH	Serum	ELISA	NR	CRP, TRAF-6, Alb, LDH, IL-1a, IL-6, TNF-α, TWEAK, orexin-A, NPY, testosterone	IL-1β, galanin
Miura *et al*. (2015) [107]	79	Participants with advanced cancer; no control	-Body composition -Fatigue	IL-6	Serum	ELISA (multiplex assay)	NR	IL-6	None
Miura *et al*. (2015) b [108]	1160	Participants with advanced cancer; no control	Survival	mGPS (Alb+CRP)	NR	NR	Primary tumor site, age and gender	mGPS (Alb+CRP)	None
Barrera *et al*. (2014) [109]	135	Participants with advanced cancer; healthy controls	-Quality of life (fatigue, PS, hyporexia, BMI) -Survival	IL-31, IL-33, IL-27, IL-29, IL-1β, IL-2, IL-6, IL-8, IL-12p70, IL-17A, IFN-γ, TNF- α, IL-4, IL-10	Plasma	CBA	No multivariate analysis	IL-6, IL-8, IFN-γ, IL-33, IL-10, IL-29^b^, IL-12p70^b^, IL17a^b^	IL-31, IL-27, IL-1β, IL-2, TNF-α, IL-4
Blakely *et al*. (2014) [110]	50	Participants with advanced cancer with normal CRP and elevated CRP	-OS -Mortality rate -gastrointestinal obstruction -Pain -Bleeding -Other symptoms (NR) -Major complications	CRP	Serum	NR	NR	CRP	None
Fujiwara *et al*. (2014) [111]	21	Participants with advanced cancer with and without cachexia	Cachexia	LP, IL-6, TNF-α	Serum	ELISA	No multivariate analysis		LP, IL-6, TNF-α
Lindemann *et al*. (2014) [112]	218	Participants with advanced cancer; no control	-Survival -Weight loss	CRP, Alb	Plasma	Immune-turbidimetry	No multivariate analysis	CRP, Alb	None
Mondello *et al*. (2014) [113]	170	Participants with advanced cancer; healthy controls	-Surviva -Cachexia	LP, ghrelin, obestatin	Serum	ELISA	Age, ghrelin, obestatin, leptin, metastatic disease and chronic kidney disease	LP, Ghrelin, obestatin	None
Moriwaki *et al*. (2014) [114]	62	Patients with advanced cancer with GPS 0, GPS 1 or GPS 2	OS	GPS (Alb+CRP), ALP, LDH, Bilirubin, CEA, CA 19-9	NR	NR	GPS, median ALP, median LDH, number of metastatic organs, liver metastasis, peritoneal metastasis, other metastasis	GPS (Alb+CRP)	ALP, Bilirubin, LDH, CEA, CA 19-9
Szkandera *et al*. (2014) [115]	474	Participants with cancer; no control	Cancer-specific survival	CRP, NLR, PLR	Plasma	NR	Age, gender, tumour grade, tumour stage, administration of chemotherapy, surgical resection, NLR, PLR, bilirubin levels and plasma CRP levels	CRP, NLR	PLR
Zhang *et al*. (2014) [116]	200	Participants with cancer; no control	-Fatigue -Chemotherapy adverse effects	TNF-α, IL-1 α, IL-1 β, 17-HCS	Plasma + urine	ELISA	No multivariate analysis	TNF-α, IL-1α, IL-1β	17-HCS
Jafri *et al*. (2013) [117]	173	Participants with advanced cancer with high inflammation and with low inflammation	-PFS -OS	ALI (Alb+NLR)	Serum	NR	Sex, race, PS and histology	ALI (Alb+NLR)	None
Laird *et al*. (2013) [118]	1466	Participants with advanced cancer with low and high CRP levels	-Symptoms of the EOTC (pain, appetite loss, cognitive function, dyspnea, fatigue, physical function, role function, social function, QoL, nausea/vomiting, diarrhea, sleep, constipation) -Survival	CRP	Blood	NR	No multivariate analysis	CRP	None
Laird *et al*. (2013) b [119]	2456	Participants with advanced cancer; no control	-Symptoms of the EOTC (pain, appetite loss, cognitive function, dyspnea, fatigue, physical function, role function, social function, QoL, nausea/vomiting, diarrhea, sleep, constipation) -Survival	mGPS (Alb+CRP)	Blood	NR	NR	mGPS (Alb+CRP)	None
Paiva *et al*. (2013) [120]	223	Participants with cancer with and without fatigue	-Fatigue -OS	CRP, Hb, LDH, Alb	Blood	NR	Age, KPS, type of treatment, breast cancer, upper gastrointestinal cancer, head and neck cancer, lower gastrointestinal cancer, lung cancer, urologic cancer, and CRP	CRP, Hb, LDH, Alb, WBC	None
Suh *et al*. (2013) [121]	98	Participants with advanced cancer; no control	Survival	IL-6, TNF-α	Plasma	ELISA (multiplex assay)	Gender (male), fatigue (BFI-K score), ECOG (3-4), IL-6 (high, ≥9.06 pg/mL)	IL-6	TNF-α
De Raaf *et al*. (2012) [122]	92	Participants with advanced cancer; cancer survivors	Physical and mental fatigue	CRP, IL-1-RA, NP, IL-6 and IL-8	Plasma	CBA	No multivariate analysis	CRP, IL-6, IL-1-ra, NP	IL-8
Gioulbasanis *et al*. (2012) [123]	114	Participants with advanced cancer with malnutrition, with a risk of malnutrition, and who were well nourished	-Nutritional status (cachexia) -Survival	IL-8	Plasma	CLIA	PS, histology, BMI, gender, age, smoking status, weight loss history	IL-8	None
Gulen *et al*. (2012) [124]	88	Participants with advanced cancer with and without weight loss; age- and sex-matched controls	Weight loss (>5%)	LP, APN, TNF-α, CRP	Serum	ELISA	No multivariate analysis	LP	APN, TNF-α, CRP
Heitzer *et al*. (2012) [125]	65	Advanced cancer patients with cancer pain; healthy controls without pain	Pain intensity	IL-1β, IL-2, IL-4, IL-5, IL-6, IL-8, IL-10, IL-12, TNF-α, TNF-β, IFN-γ, IL-1α, IL-7, IL-13, IL-18, MCP-1, MIP-1a, MIP-1B, OPG	Serum	ELISA	NI	Unclear	Unclear
Minton *et al*. (2012) [126]	720	Participants with advanced cancer with and without fatigue	Fatigue	CRP, Alb, Hb	Blood	NR	Hb, current treatment with chemo, QOL score, depression, pain dyspnoea, cognitive function, insomnia and loss of appetite	CRP, Alb, Hb	None
Partridge *et al*. (2012) [127]	102	Patients with advanced cancer with GPS 0, GPS 1 or GPS 2 ; no control	Survival	mGPS (Alb+CRP)	Blood	NR	Sex, primary cancer site, age, Hb and WBC	mGPS (Alb+CRP)	None
Pond *et al*. (2012) [128]	220	Participants with advanced cancer; no control	-OS -PFS	CRP	NR	NR	NR	CRP	None
Wang *et al*. (2012) [129]	177	Participants with cancer; no control	Survival	CRP, Alb, mGPS (Alb+CRP), NLR	NR	NR	PS, pretherapeutic weight, WBC, neutrophil count, NLR, CRP, mGPS, PI, the 7^th^ TNM staging, surgery, degree of differentiation, palliate chemotherapy	CRP, mGPS (Alb+CRP), NLR	Alb
Aydin *et al*. (2011) [130]	61	Advanced cancer patients; no control	Survival	CRP, Alb, TFN	Serum	Nephelometric assay	No multivariate analysis	CRP, Alb, TFN	None
Dev *et al*. (2011) [131]	77	Participants with advanced cancer; no control	Symptom distress (pain, fatigue, nausea, depression, anxiety, drowsiness, appetite, well-being, dyspnea, sleep)	Cortisol	Serum	NR	NR	Cortisol	None
Gioulbasanis *et al*. (2011) [132]	115	Participants with advanced cancer with malnutrition, with a risk of malnutrition, and who were well nourished	-Nutritional status (cachexia) -Survival	Alb, CRP, ghrelin, LP, APN, IGF-1	Plasma	Radioimmunoassay	Number of metastatic sites, PS, weight loss <5%, MNA groups, age, and major histological type	CRP, LP, Alb	Ghrelin, APN, IGF-1
Hwang *et al*. (2011) [133]	402	Participants with cancer; no control	-PFS -OS	Alb, CRP	Serum	Latex turbidimetric immunoassay	Peritoneal metastasis, bone metastasis, albumin, CRP, ECOG PS, GPS	Alb, CRP	None
Kwak *et al*. (2011) [134]	90	Participants with advanced cancer; no control	Fatigue	IL-6, TNF-α	Blood	NR	BFI score, age, gender, BMI, blood pressure, heart rate, cancer site, previous treatment, comorbidity, medication, pain score, sleep disorder, dyspnea, ECOG PS, WBC, Hb, BUN, creatinine, albumin, AST, ALT, total bilirubin, CRP, IL-6, and TNF-α	None	IL-6, TNF-α
Lee *et al*. (2011) [135]	126	Participants with advanced cancer; no control	14 day mortality	CRP	Serum	NR	CRP, chemotherapy, age, dyspnea, altered mental status, hypotension, and leukocytosis	CRP	None
Scheede-Bergdahl *et al*. (2011) [136]	83	Participants with advanced cancer; no control	- Clinical features of cachexia (weakness, loss of appetite, fatigue, QOL, weight loss) -Survival	IL- 6, IL-1β, IL-8, TNF-α	Plasma	BCA	Sex, age, diagnosis, oncological treatment, CCI and medications	IL- 6, IL-1β, IL-8, TNF-α	None
Vlachostergios *et al*. (2011) [137]	77	Participants with advanced cancer; no control	-TTP -OS	IGF-1, CRP, Alb	Serum	Radioimmunoassay	Sex, current smoker, albumin, IGF-1	IGF-1, CRP, Alb	None
Diakowska *et al*. (2010) [138]	218	Participants with cancer with and without cachexia; healthy blood donors; and patients with non-malignant diseases of alimentary tract	Cachexia	LP, CRP, IL-1, IL-6, IL-8, TNF-α, Alb, Hb.	Serum	ELISA	NR	LP, IL-6, Alb, TNF-α	IL-1, IL-8, Hb, CRP*
Meek *et al*. (2010) [139]	56	Participants with advanced cancer; no control	Cancer-specific survival	IGF-1, IGFBP-3, CRP, mGPS (Alb+CRP), LP	Serum	NR	BMI, cancer stage, Hb, WBC, mGPS	mGPS (Alb+CRP)	IGF-1, IGFBP-3, LP, CRP
Ishizuka *et al*. (2009) [140]	112	Participants with advanced cancer; no control	Mortality	CRP, Alb, mGPS (Alb+CRP), Neutrophil ratio	Serum	NR	Neutrophil ratio, CA 19–9, CRP, albumin, and mGPS	mGPS (Alb+CRP)	None
Karapanagiotou *et al*. (2009) [141]	161	Participants with advanced cancer; healthy controls	-Weight loss -TTP -OS	Ghrelin, LP	Serum	ELISA	Sex, age, BMI, Ghrelin	Ghrelin Multivariate results NR	LP Multivariate results NR
Paddison *et al*. (2009) [142]	44	Participants with advanced cancer; healthy controls	Fatigue	Hb, WBC, Neutrophil, Monocyte,Lymphocyte	Blood	NR	Age, gender, time until treatment termination; and fatigue	Hb, WBC, Neutrophil count, monocyte count	None
Takahashi *et al*. (2009) [143]	26	Participants with cancer cachexia; healthy controls	Anorexia (cachexia and BMI)	TNF-α, IFN-γ, IL-6, IL-1RA, LP, ghrelin	Plasma	ELISA	No multivariate analysis	TNF-α, IL-6, IL-1RA, LP	IFN- γ, ghrelin
Inagaki *et al*. (2008) [144]	46	Participants with advanced cancer with and without fatigue	Fatigue	IL-6	Plasma	ELISA	Logistic regression: IL-6, gender, weight and clinical fatigue Multiple regression: gender, weight, IL-6 and total score of the CFS	IL-6	None
Karapanagiotou *et al*. (2008) [145]	152	Participants with advanced cancer; healthy controls	-Weight loss -TTP -OS	LP, APN, resistin	Serum	ELISA	Sex, age, BMI, resistin	Resistin	LP, APN
Sharma *et al*. (2008) [146]	52	Participants with advanced cancer; no control	-OS -Toxicity	IL-1β, IL-2, IL-4, IL-5, IL-8, IL-6, IL-10, IL-12, GM-CSF, IFN-Y, TNF-α, sIL-6R, sgp130, VEGF, eotaxin, MCP-1, MIP-1α, MIP-1β, Alb, CRP, GPS (Alb+CRP)	Serum	NR	Tumour site (colonic primary), GPS, CEA, and albumin	GPS (Alb+CRP), Hb, Alb	CRP, IL-1β, IL-2, IL-4, IL-5, IL-8, IL-6, IL-10, IL-12, GM-CSF, IFN-Y, TNF-α, sIL-6R, sgp130, VEGF, eotaxin, MCP-1, MIP-1α, MIP-1β
Weryńska *et al*. (2008) [147]	40	Participants with advanced cancer with and without cachexia	-Cachexia -Nutritional status	LP	Serum	ELISA	No multivariate analysis	LP	None
Ravasco *et al*. (2007) [148]	101	Participants with cancer; no control	-REE -Weight loss -Nutritional intake	IL-1RA, IL-6, TNF-α, IL-10, IFN-γ, VEGF	Serum	ELISA	Cancer histology and stage, nutritional intake	IL-1RA, IL-6, TNF-α, IFN-y, VEGF	IL-10
Richey *et al*. (2007) [149]	24	Participants with cancer with and without cachexia	Cachexia	GPS (Alb+CRP), Alb, IL-1a, IL-1β, IL-2, IL-4, IL-5, IL-6, IL-8, IL-10, IL-12, TNF-α, IFN-γ, VEGF, GM-CSF, MCP-1, MIP-1a, MIP-1B, RANTES, FGF, Hb, CRP, CEA	Serum	Dry-slide method with the VITROS Fusion Series analyser	No multivariate analysis	GPS (Alb+CRP), Alb, CEA	IL-1a, IL-1β, IL-2, IL-4, IL-5, IL-6, IL-8, IL-10, IL-12, TNF-α, IFN-y, VEGF, GM-CSF, GM-CSF, MCP-1, MIP-1a, MIP-1B, RANTES, FGF, Hb, CRP, CEA
Suh *et al*. (2007) [150]	44	Participants with advanced cancer; no control	Survival	CRP	Serum	NR	NR	CRP	None
Al Murri *et al*. (2006) [151]	96	Breast cancer patients; no control	Survival	CRP, Alb, GPS (Alb+CRP)	NR	NR	GPS and treatment	CRP, GPS (Alb + CRP)	None
Kayacan *et al*. (2006) [152]	56	Participants with advanced cancer with and without cachexia; healthy smokers for the control	-Cachexia -PS -Survival	TNF-α, IL-6	Serum	ELISA	NR	None	TNF-α, IL-6
Ramsey *et al*. (2006) [153]	119	Participants with advanced cancer; no control	-Cancer-specific survival -Cancer-specific mortality	GPS (Alb+CRP)	NR	NR	GPS, Hb, calcium, WBC, neutrophil count, Alb, CRP	GPS (Alb+CRP)	None
Di Nisio *et al*. (2005) [154]	141	Participants with advanced cancer; no control	Survival	IL-6, IL-10, IFN-y, P-selectin	Plasma	BCA	Life expectancy, WHO performance status, concomitant treatment, type of carcinoma, and histology	IL-10, IL-6, P-selectin	IFN-y
Rich *et al*. (2005) [155]	80	Participants with advanced cancer with good and dampened circadian rhythms	-Extent of metastatic disease -PS -QOL	IL-6, TGF-a, TNF-α, cortisol	Serum	ELISA	NR	IL-6, TGF-a, TNF-α	Cortisol
Bolukbas *et al*. (2004) [156]	69	Participants with advanced cancer; healthy controls with stable weight	Weight loss	LP	Serum	ELISA	NR	LP	None
De Vita *et al*. (2004) [157]	68	Participants with advanced cancer; no control	-TTP -OS	IL-6	Serum	ELISA	NR	Il-6	None
Dulger *et al*. (2004) [158]	54	Participants with advanced cancer with and without cachexia; healthy gender- and age- matched adults	Cachexia	TNF-α, IL-1β, IL-6, CRP, LP, GH, TG, insulin, glucose, triglyceride, total protein, ESR	Serum	Solid-phase, two-site chemiluminescent immunometric assays	No multivariate analysis	Alb, total protein, GH, TNF-α, IL-1β, IL-6, insulin, LP, ESR^b^, CRP^b^	Glucose, TG
Elahi *et al*. (2004) [159]	165	Participants with advanced cancer; no control	Survival	Alb, CRP	NR	Fluorescence polarization immunoassay	NR	Alb, CRP	None
Jamieson *et al*. (2004) [160]	33	Participants with advanced cancer; healthy controls	Weight loss	Hb, Alb, CRP, APN, LP, IL-6	Serum	ELISA	No multivariate analysis	Hb, Alb, CRP, APN, LP, IL-6	None
Songur *et al*. (2004) [161]	91	Participants with advanced cancer; healthy controls	-Malnutrition -Survival	IL-6, Alb, CRP, TFN, LDH	Serum	NR	NR	IL-6, Alb, CRP, TFN, LDH	None
Scott *et al*. (2003) [162]	106	Participants with advanced cancer with and without weight loss	-Weight loss	Hb, Alb, CRP	Blood	NR	No multivariate analysis	Hb, Alb, CRP	None
Aleman *et al*. (2002) [163]	106	Patients newly diagnosed with NSCL vs patients with no cancer	-Nutritional status -Survival	IL-6, IL-12, IL-10, IL-2, LP, α -1A, ferritin, CRP, TNF-α, s-TNFR2, s-IL-2R, IFN-γ	Serum	CLIA	NR	IL-6, IL-12, IL-2, sTNFR2, IFN-γ, sIL-2R, LP, α-1A, CRP, ferritin Multivariate results unclear	IL-10, TNF-α Multivariate results unclear
Orditura *et al*. (2002) [164]	85	Participants with advanced cancer; healthy controls	-OS -TTF	IL-8, IL-10, IL-2	Serum	ELISA	NR	IL-10, IL-2, IL-8	None
Scott *et al*. (2002) [165]	106	Participants with advanced cancer; no control	Survival	Alb, CRP	Blood	NR	Age, sex, stage, histological type, weight loss, haemoglobin, albumin, CRP, KPS and EORTCV QLQ-C30 subscale	CRP, Alb	None
Jatoi *et al*. (2001) [166]	73	Participants with advanced cancer; healthy controls	Anorexia and/or weight loss	NPY, LP, CCK-8	Serum	Radioimmunoassay	No multivariate analysis	NPY	LP, CCK-8
Mantovani *et al*. (2001) [167]	58	Participants with advanced cancer; normal weight healthy controls	-BMI -Cachexia -ECOG PS -Survival	LP, IL-6, TNF-α	Serum	ELISA	No multivariate analysis	Unclear	Unclear
Mantovani *et al*. (2000) [168]	32	Participants with advanced cancer; normal weight healthy controls	-cachectic symptoms (BMI)	LP, IL-1a, IL-6, and TNF-α	Serum	ELISA	No multivariate analysis	Unclear	Unclear
Nenova *et al*. (2000) [169]	87	Participants with advanced cancer; healthy controls	-Cachexia -Prognosis	TNF-α	Serum	ELISA	No multivariate analysis	Unclear	Unclear
O'Gorman *et al*. (1999) [170]	50	Participants with advanced cancer with weight loss or weight gain; weight stable controls	-Weight loss -Appetite -PS -Inflammation	Alb, CRP	Blood	NR	No multivariate analysis	Alb, CRP	None
Okada *et al*. (1998) [171]	100	Participants with cancer; healthy controls	Weight loss	IL-6	Serum	ELISA	No multivariate analysis	IL-6	None
Wallace *et al*. (1998) [172]	54	Participants with advanced cancer; healthy controls	Weight loss	LP	Serum	Radioimmunoassay	No multivariate analysis	LP	None
Maltoni *et al*. (1997) [173]	530	Participants with advanced cancer; no control	Survival	Neutrophil, lymphocyte & monocyte %, basophil + eosinophil %, Hb, TFN, Alb, total WBC, Pseudocholinesterase, proteinuria, TFN, transport iron	Blood	NR	No multivariate analysis	Neutrophil %, lymphocyte %, total WBC, CHE, Alb	basophil + eosinophil %, Hb, TFN
Simons *et al*. (1997) [174]	21	Participants with cancer and weight loss; no control	-Weight loss -Body composition -Appetite -REE	LP	Plasma	ELISA	No multivariate analysis	LP	None

Note: Cancer prognosis was not separated from the other syndromes in the table

* Red coloured biomarkers indicate significance in multivariate analysis

^a^Secondary analysis of Amano, 2016

^b^In cancer vs no cancer only

Abbreviations: *17-HCS* 17-hydroxycorticosteroids, *α-1-AGP* a-1-acid glycoprotein, *α-1A* alpha-1 antitrypsin, *Alb* Albumin, *ALP* Alkaline phosphatase, *APN* Adiponectin, *APOA2* Apolipoprotein A2, *BCA* The bicinchoninic acid assay, *bFGF* Basic fibroblast growth factor, *CA 19-9* Cancer antigen, *CBA* Cytometric bead array immunoassay, *CCK* Cholecystokinin, *CEA* Carcinoembryonic antigen, *CK* Creatine Kinase, *CLIA* Chemiluminescence immunoassay, *Cre* Creatinine, *CRP* C-Reactive Protein, *CXCL* Soluble CXC chemokine ligand, *ESR* Erythrocyte sedimentation rate, *FBG* Fibrinogen, *FSN* Follistatin, *GH* Growth Hormone, *GM-CSF* Granulocyte-Macrophage Colony-Stimulating Factor, *HA* Hyaluronic Acid, *Hb* Haemoglobin, *IGF* Insulin-Like Growth Factor, *IGFBP* Insulin-like Growth Factor Binding Protein, *IL* Interleukin, *IFN* Interferon, *LDH* Lactate Dehydrogenase, *LP* Leptin, *MCP* Monocyte Chemotactic Protein, *MIP* Macrophage Inflammatory Protein, *MK* Midkine, *NI* Not enough information, *NR* Not reported, *MSTN* Myostatin, *NLR* Neutrophil-lymphocyte ratio, *NP* Neopterin, *NPY* Neuropeptide Y, *OPG* Osteoprotegrin, *PLR* Platelet-lymphocyte ratio, *RANTES* Chemokine (C-C motif) ligand 5, *sTNFR* SolubleTumor Necrosis Factor Receptor, *Sgp130* Soluble **glycoprotein** 130, *TARC* Thymus and Activation-Regulated Chemokine, *TFN* Transferrin, *TG* Triglyceride, *TNF* Tumor Necrosis Factor, *TRAF-6* Tumor Necrosis Factor Receptor associated factor-6, *TTF* Time to treatment failure, *TWEAK* TNF-like weak inducer of apoptosis, *VEGF* Vascular Endothelial Growth Factor, *ZAG* Zn-alpha2 glycoprotein

**Fig. 2:**
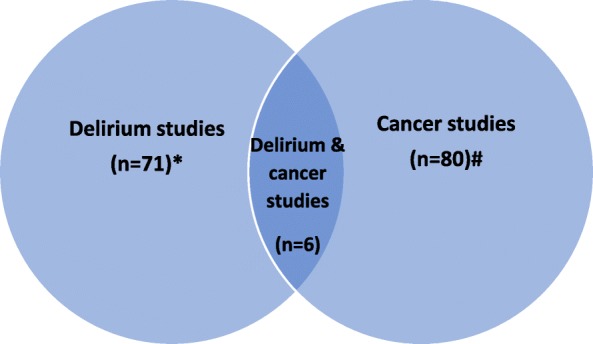
Conceptual model illustrating the ‘true overlap’ of delirium and advanced cancer biomarker studies. * Cancer as a comorbidity not measured/reported # Delirium as a concurrent illness or comorbidity not measured/reported

A total of 41 biomarkers were found to be common in both delirium and advanced cancer syndrome studies. The five most commonly studied biomarkers were C-reactive protein (CRP) (n=79), interleukin (IL)-6 (*n*=58), tumor necrosis factor alpha (TNF- α) (*n*=42) IL-10 (*n*=21) and IL-8 (*n*=24). Of these, 24 biomarkers had a positive association with delirium, cancer prognosis or a cancer syndrome in at least one study. No cancer studies reported having any participants with delirium, and of the delirium studies, six reported participants with cancer. Figure 2 illustrates two main populations identified from this systematic review, with the centre showing the ‘true overlap’ defined as studies that included participants with both delirium and cancer (n=6 studies).

In two of these studies, all participants in the study had cancer; in another, 64.2% of participants had cancer; in the remaining three studies, less than 30% of all participants had cancer. In three of the studies, 100% of participants who had delirium also had cancer, in another two, 26% and 27% of the delirium cohorts had cancer, and in the remaining study 14% of the delirium participants had cancer (Table 1). Although only six delirium studies reported co-existing cancer, there is still uncertainty as to how many participants in both groups of studies had both delirium and cancer. The two most common biomarkers in these six studies that reported a positive association with delirium were CRP (*n*=3) and IL-6 (*n*=3). It is unclear however whether these biomarkers were predominantly associated with delirium or the cancer, as three of the six studies grouped the delirium participants together, irrespective of their cancer comorbidity.

The quality assessment showed a large variability in the reporting of included studies. 150 (99%) studies had a clear aim statement which included their outcome of interest. One study did not report a clear aims statement [175]. One hundred and nineteen studies (79%) did not explicitly state the hypothesis; however, in most (n=94; 62%) the hypothesis could be interpreted by the study aim. All 151 studies stated the participant population in detail. No study reported all elements of the assay methods in the REMARK checklist [23]. One hundred and thirty one studies (87%) did not report whether assays were blinded to the study endpoint, however 59 (45%) of those studies were objective assessments. Further, 14 studies (9%) reported a power calculation to justify their sample size. Most (n=125; 83%) of studies defined all clinical endpoints examined. Ninety seven (64%) studies undertook multivariate analysis, and of these 67 (69%) described the multivariate model and the covariates included in the model, and 23 (23%) explained the rationale for inclusion of the covariates in the models. (Additional files 4 and 5). Furthermore, 27 delirium studies (38%) did not report the reason for admission. Of the 44 studies that did report the reason for admission, these were predominantly for surgery- elective and acute (*n*=40). Most studies in the non-surgical population did not report a reason for admission, with the exception of 4 studies where the medical condition of interest occurred on admission (e.g stroke). See additional files 4 and 5 for the complete quality assessments.

**Fig. 3:**
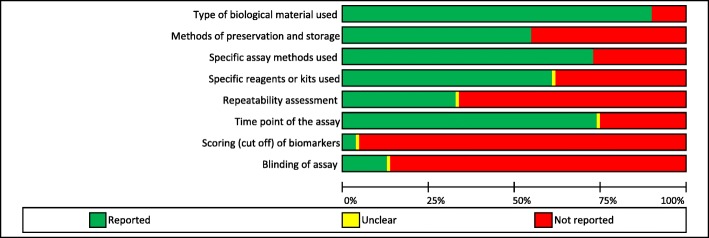
Quality assessment graph of the assay procedures: review author’s judgements about each assay domain of the REMARK checklist, presented as percentages across studies

The methodological quality of the assay procedures only is depicted in Figure 3, with reporting of type of biological material mostly provided but much lower frequency of reporting for other critical descriptors.

## Discussion

This is the first systematic review to our knowledge, to demonstrate the high degree of overlap in biomarkers in delirium, cancer prognosis and advanced cancer syndromes. This systematic review of 151 studies found that 41 biomarkers were independently investigated in studies of both delirium and prognosis/advanced cancer syndromes; with over half having a positive association in at least one study.

Biomarkers fall into three categories (though not mutually exclusive); those which present before disease onset that can help identify individuals who are most at risk of a particular disease (for example, genetic markers), those which are disease markers and as such, increase during disease progression and decrease after resolution, and thirdly, biomarker as an end-product of a disease for which levels are proportionate to ‘damage’ due to the disease [176]. The findings of this systematic review suggest that categorization along these lines is less understood in delirium. For example, there is evidence to show that conditions such as sepsis and hip fracture cause changes in inflammatory markers [177, 178], however, there is little evidence about whether delirium self-propagates. Some animal model data in delirium suggests that there might be a direct impact of inflammatory markers on brain dysfunction [179]. To our knowledge there was no published relationship between tumor markers and neurological brain dysfunction. Although clinical evidence suggests long term impacts on brain function, the exact pathophysiological mechanisms are poorly understood, and biomarkers to measure this are also unclear.

The issue of biomarker overlap between associated conditions has been researched in women with pre-eclampsia and polycystic ovary syndrome [180], however the overlap with respect to delirium and its associated conditions has not been well addressed. Of the 71 delirium studies, only five studies sought to determine the association with the participants’ common primary condition in their analysis. Tomasi et al. (2017) found that biomarkers differed between patients in the three groups in those with sepsis alone and those who developed sepsis-associated encephalopathy, or delirium, suggesting different mechanisms of sepsis-associated encephalopathy, delirium in people with sepsis, and sepsis itself. Likewise, Pfister et al. (2008) found differences in CRP, s100 calcium binding protein B (s100B) and cortisol in patients with sepsis-associated delirium, compared to non-sepsis associated delirium. In two studies, delirium in stroke was examined [25, 92] but these studies did not identify differences in cortisol [92] or TNF- α, IL- 1β, IL-18, Brain-derived neurotrophic factor (BDNF) and Neuron specific enolase (NSE) [25] between patients who developed delirium after stroke compared to those who did not develop delirium. Moreover, Sun et al. (2016) attempted to explore the overlap of biomarkers in delirium and dementia in patients with cancer, however, no multivariate analysis was undertaken, therefore results of this study are inconclusive.

Although the aim of this systematic review was to explore the overlap of biomarkers in delirium and advanced cancer syndromes, the findings highlighted a bigger problem in the methodology of delirium biomarker research. The quality assessment in this systematic review found that many of the included studies were of poor methodological quality, inadequately reported, or were influenced by potential confounding factors. A potential barrier to the complete understanding of delirium pathophysiology is the lack of guidelines for conducting and reporting delirium biomarker studies. Results from this review indicate that the absence of such guidelines has likely impeded the quality of individual studies and the overall quality of this critical field of delirium research. Reporting guidelines for delirium biomarker research are an essential step to improving methodological and reporting rigor, and will increase the potential for synthesis of future studies through meta-analyses.

Several studies have previously been performed to determine biomarkers associated with delirium, however potential confounding factors could be the underlying precipitants of delirium; ie risk factors (sepsis), or underlying conditions present (for example cancer or dementia). The top five most commonly studies biomarkers in this review were inflammatory biomarkers, namely, CRP, IL-6, TNF- α, IL-10 and IL-8. The challenge with inflammatory markers is that they are non-specific and the inflammatory pathways are similar to those implicated in other conditions such as sepsis and depression [181, 182]. Likewise, of the six delirium studies where there was concomitant cancer, it is very difficult to determine whether those biomarkers found were related to the cancer or the delirium itself, considering alterations in inflammatory pathways are implicated in both. Therefore, future delirium biomarker studies need to be prospectively evaluated and take into account and assess robustly other active co-morbidities such as cancer that could plausibly impact on the pathophysiological and/or biological findings. Similarly, future cancer biomarker studies must also take into account how delirium may clinically or biologically confound biomarker studies in cancer, considering the high prevalence of delirium in this population. Of the six delirium studies with cancer, three did not report the type of cancer, and of the remaining three studies, none were primary brain tumours or brain metastases. Understanding the spread of brain cancer is important in delirium studies, and is an important consideration for future delirium biomarker studies.

Majority of the studies in this review (n=98; 65%) undertook a multivariate analysis, taking into account confounding variables. Where studies only undertook univariate analysis, it is uncertain whether any observed changes in biomarkers were related to the delirium itself, or whether these changes may have been lost when adjusted for confounding factors (such as prior cognitive impairment) in a multivariate analysis. Furthermore, there is likely to be a higher proportion of participants with both delirium and cancer in both groups of studies for which this clinical information was not assessed or that were not reported. Key methodological issues which need to be addressed in future delirium studies include adjusting for confounders such as age, gender, concurrent medication, comorbidities, prior cognitive impairment, frailty and other neurological conditions. These clinical covariates must also be clearly defined and justified. Assay procedures ought to be reported in detail, including a detailed protocol of the reagents/kits used, repeatability assessments, methods of preservation and storage, assay validity, sensitivity limits of the assay and a scoring and reporting protocol. The timing of the assay is crucial in delirium studies, and the fluctuating pathophysiological processes occurring during delirium, after delirium resolution, and in those who have not yet developed delirium, must be taken into consideration, and be separated in future studies. More standardised and detailed methods of delirium biomarker studies is a crucial step in carrying out future subgroup analyses within this cohort and improving the overall understanding of delirium pathophysiology.

Limitations are that only English language and published studies were included. It is possible that articles were missed; however, two reviewers independently screened all citations derived from a search of six relevant and diverse databases, and all reference lists of included articles were also searched. Another limitation of our study is the lack of a risk of bias tool for biomarker studies, therefore we used an adaptation of tumor marker reporting guidelines, the REMARK checklist [23]. Lastly, the heterogeneity of the data precluded the conduct of a meta-analysis, and precluded any firm conclusions about the biomarkers in delirium and cancer, thus, limiting the rigor of this review. Strengths of this review however, were that we undertook a systematic approach adhering to the PRISMA [15] and an extensive quality assessment of the included studies was undertaken.

## Conclusion

This review found that there is large overlap in the biomarkers in delirium and in advanced cancer-related syndromes, although because of the heterogeneity of the studies firm conclusions about the true overlap of delirium and advanced cancer syndrome biomarkers was not possible. More robust conduct and reporting of delirium biomarker studies will help to better understand the pathophysiology of delirium in the context of co-existing pathophysiology. An improved understanding of the clinical and biological associations of delirium and advanced cancer syndromes in future prospective studies will provide and inform the directions of research into delirium in people with advanced cancer.

## Supplementary information

**Additional file 1:.** MEDLINE search strategies MEDLINE search strategies for delirium and cancer studies.

**Additional file 2:.** Participant characteristics- delirium studies Characteristics of participants in the included delirium studies.

**Additional file 3:.** Participant characteristics- cancer studies Characteristics of participants in the included cancer studies.

**Additional file 4:.** Quality assessment of included delirium studies using the REMARK checklist The quality assessment for all included delirium studies.

**Additional file 5:.** Quality assessment of included cancer studies using the REMARK checklist The quality assessment for all included cancer studies.

**Additional file 6:.** PRISMA checklist.

## Data Availability

All data generated or analysed in this systematic review are included within this published article and its additional files.

## Abbreviations

BDNF: Brain-derived neurotrophic factor
CRP: C-reactive protein
CSF: Cerebrospinal fluid
ELISA: Enzyme-linked immunosorbent assay
IL-: Interleukin
NSE: Neuron specific enolase
S100B: S100B calcium binding protein B
TNF: Tumor necrosis factor
