# Supplementary material for: A systematic review of the overlap of fluid biomarkers in delirium and advanced cancer-related syndromes
Source: BMC Psychiatry. 2020 Apr 22;20:182. doi: 10.1186/s12888-020-02584-2 (PMC7178636; doi:10.1186/s12888-020-02584-2)
Supplement: Supplementary file 1 — Additional file 1:. MEDLINE search strategies MEDLINE search strategies for delirium and cancer studies. [file 12888_2020_2584_MOESM1_ESM.docx]

| **MEDLINE- Delirium** | | |
| --- | --- | --- |
| **#** | **Searches** | **Results** |
| 1 | delirium.m_titl. | 6535 |
| 2 | "delir*".m_titl. | 6847 |
| 3 | "acute confusion".m_titl. | 122 |
| 4 | "acute organic psychosyndrome".m_titl. | 4 |
| 5 | "acute brain syndrome".m_titl. | 23 |
| 6 | "metabolic encephalopathy".m_titl. | 76 |
| 7 | "acute psycho-organic syndrome".m_titl. | 3 |
| 8 | "clouded state".m_titl. | 2 |
| 9 | "clouding of consciousness".m_titl. | 18 |
| 10 | "exogenous psychosis".m_titl. | 15 |
| 11 | "toxic psychosis".m_titl. | 106 |
| 12 | "toxic confusion".m_titl. | 2 |
| 13 | 1 or 2 or 3 or 4 or 5 or 6 or 7 or 8 or 9 or 10 or 11 or 12 | 7207 |
| 14 | Biomarkers/ or biomarker*.mp. | 423459 |
| 15 | Cytokines/ or cytokine*.mp. | 340463 |
| 16 | tryptophan.mp. or Tryptophan/ | 54367 |
| 17 | melatonin.mp. or Melatonin/ | 22624 |
| 18 | serotonin.mp. or Serotonin/ | 138213 |
| 19 | chemokine*.mp. | 78017 |
| 20 | interleukin.mp. or Interleukins/ | 302129 |
| 21 | S100 Proteins/ or S100b.mp. or S100 Calcium Binding Protein beta Subunit/ | 12404 |
| 22 | cortisol.mp. | 54671 |
| 23 | "S100 beta".mp. | 251 |
| 24 | "TNF alpha".mp. or Tumor Necrosis Factor-alpha/ | 159040 |
| 25 | IGF-1.mp. or Insulin-Like Growth Factor I/ | 37447 |
| 26 | "apolioprotein E4".mp. or Apolipoproteins E/ | 16307 |
| 27 | "C reactive protein".mp. or C-Reactive Protein/ | 64777 |
| 28 | C-Reactive Protein/ or CRP.mp. | 57755 |
| 29 | Dopamine/ or dopamine.mp. | 146886 |
| 30 | neurotransmitter*.mp. | 86313 |
| 31 | 14 or 15 or 16 or 17 or 18 or 29 or 20 or 21 or 22 or 23 or 24 or 25 or 26 or 27 or 28 or 29 or 30 | 86313 |
| 32 | 13 and 31 | 998 |
| 33 | limit 32 to (yr="1980 -Current" and english and humans) | 703 |

| **MEDLINE- cancer prognosis** | | | | |
| --- | --- | --- | --- | --- |
| **#** | | **Searches** | **Results** | |
| 1 | | (cancer adj5 prognosis).m_titl | 6670 | |
| 2 | | prognostication.mp. | 5636 | |
| 3 | | 1 or 2 | 12260 | |
| 4 | | cancer.mp. or Neoplasms/ | 2831489 | |
| 5 | | Advanced.mp. | 381443 | |
| 6 | | metastasis.mp. or Neoplasm Metastasis/ | 325957 | |
| 7 | | end stage".mp. | 63359 | |
| 8 | | "late stage".mp. | 19595 | |
| 9 | | "stage 4".mp. | 5379 | |
| 10 | | "stage four".mp. | 258 | |
| 11 | | 5 or 6 or 7 or 8 or 9 or 10 | 853407 | |
| 12 | | Biomarkers/ or biomarker*.mp. | 330208 | |
| 13 | | Cytokines/ or cytokine*.mp. | 533139 | |
| 14 | | tryptophan.mp. or Tryptophan/ | 68521 | |
| 15 | | melatonin.mp. or Melatonin/ | 32995 | |
| 16 | | serotonin.mp. or Serotonin/ | 203499 | |
| 17 | | chemokine*.mp. | 117568 | |
| 18 | | interleukin.mp. or Interleukins/ | 555852 | |
| 19 | | S100 Proteins/ or S100b.mp. or S100 Calcium Binding Protein beta Subunit/ | 24409 | |
| 20 | | cortisol.mp. | 67889 | |
| 21 | | "S100 beta".mp. | 175 | |
| 22 | | "TNF alpha".mp. or Tumor Necrosis Factor-alpha/ | 233733 | |
| 23 | | IGF-1.mp. or Insulin-Like Growth Factor I/ | 52614 | |
| 24 | | "apolipoprotein E".mp. or Apolipoproteins E/ | 23229 | |
| 25 | | "C reactive protein".mp. or C-Reactive Protein/ | 137997 | |
| 26 | | C-Reactive Protein/ or CRP.mp. | 156134 | |
| 27 | | Dopamine/ or dopamine.mp. | 203307 | |
| 28 | | neurotransmitter*.mp. | 100938 | |
| 29 | | 12 or 13 or 14 or 15 or 16 or 17 or 18 or 19 or 20 or 21 or 22 or 23 or 24 or 25 or 26 or 27 or 28 | 1743254 | |
| 30 | | 3 and 4 and 11 and 29 | 328 | |
| 31 | | limit 30 to (yr="1980 -Current" and English and humans) | 251 | |
| **MEDLINE- Anorexia cachexia** | | | | |
| [**#**](http://ovidsp.tx.ovid.com.ezproxy.lib.uts.edu.au/sp-3.18.0b/ovidweb.cgi?&S=LAIPFPPIBODDMAHBNCJKJELBHKLIAA00&Sort+Sets=descending) | **Searches** | | | **Results** |
| 1 | Cachexia/ or "anorexia cachexia".mp. | | | 4814 |
| 2 | cachexic.mp. | | | 83 |
| 3 | wasting syndrome/ | | | 1106 |
| 4 | (anorexia adj5 cachexia).mp. | | | 875 |
| 5 | 1 or 2 or 3 or 4 | | | 6046 |
| 6 | Biomarkers/ or biomarker*.mp. | | | 344307 |
| 7 | Cytokines/ or cytokine*.mp. | | | 533139 |
| 8 | tryptophan.mp. or Tryptophan/ | | | 68521 |
| 9 | melatonin.mp. or Melatonin/ | | | 32995 |
| 10 | serotonin.mp. or Serotonin/ | | | 203499 |
| 11 | chemokine*.mp. | | | 117568 |
| 12 | interleukin.mp. or Interleukins/ | | | 555852 |
| 12 | IL.mp. or Interleukins/ | | | 423394 |
| 13 | S100 Proteins/ or S100b.mp. or S100 Calcium Binding Protein beta Subunit/ | | | 24409 |
| 14 | cortisol.mp. | | | 67889 |
| 15 | "S100 beta".mp. | | | 175 |
| 16 | "TNF alpha".mp. or Tumor Necrosis Factor-alpha/ | | | 233733 |
| 17 | IGF-1.mp. or Insulin-Like Growth Factor I/ | | | 52614 |
| 17 | Apolipoproteins E/ or "apolipoprotein E".mp. | | | 28815 |
| 18 | "C reactive protein".mp. or C-Reactive Protein/ | | | 137997 |
| 19 | “CRP”.mp. | | | 70815 |
| 20 | Dopamine/ or dopamine.mp. | | | 203307 |
| 22 | neurotransmitter*.mp. | | | 100938 |
| 23 | 6 or 7 or 8 or 9 or 10 or 11 or 12 or 13 or 14 or 15 or 16 or 17 or 18 or 19 or 20 or 21 or 22 | | | 2041019 |
| 24 | cancer.mp. or Neoplasms/ | | | 2831489 |
| 25 | Advanced.mp. | | | 347554 |
| 26 | metastasis.mp. or Neoplasm Metastasis/ | | | 301151 |
| 27 | "end stage".mp. | | | 57570 |
| 28 | "late stage".mp. | | | 17564 |
| 29 | "stage 4".mp. | | | 4931 |
| 30 | "stage four".mp. | | | 237 |
| 31 | 25 or 26 or 27 or 28 or 29 or 30 | | | 694442 |
| 32 | 5 and 23 and 24 and 31 | | | 1409 |
| 34 | limit 32 to (yr="1980 -Current" and english and humans) | | | 468 |
| **MEDLINE- cognitive impairment** | | | | |
| [**#**](http://ovidsp.tx.ovid.com.ezproxy.lib.uts.edu.au/sp-3.18.0b/ovidweb.cgi?&S=LAIPFPPIBODDMAHBNCJKJELBHKLIAA00&Sort+Sets=descending) | | **Searches** | **Results** | |
| 1 | | “chemo brain” | 47 | |
| 2 | | “chemo fog” | 23 | |
| 3 | | "cognitive impairment".mp. or Cognitive Dysfunction/ | 42832 | |
| 4 | | 1 or 2 or 3 | 42874 | |
| 5 | | cancer.mp. or Neoplasms/ | 1574769 | |
| 6 | | Advanced.mp. | 381443 | |
| 7 | | metastasis.mp. or Neoplasm Metastasis/ | 325957 | |
| 8 | | "end stage".mp. | 63359 | |
| 9 | | "late stage".mp. | 19595 | |
| 10 | | "stage 4".mp. | 5379 | |
| 11 | | "stage four".mp. | 258 | |
| 12 | | 6 or 7 or 8 or 9 or 10 or 11 | 757866 | |
| 13 | | Biomarkers/ or biomarker*.mp. | 426688 | |
| 14 | | Cytokines/ or cytokine*.mp. | 340463 | |
| 15 | | tryptophan.mp. or Tryptophan/ | 54367 | |
| 16 | | melatonin.mp. or Melatonin/ | 22624 | |
| 17 | | serotonin.mp. or Serotonin/ | 138213 | |
| 18 | | chemokine*.mp. | 78017 | |
| 19 | | interleukin.mp. or Interleukins/ | 302129 | |
| 20 | | S100 Proteins/ or S100b.mp. or S100 Calcium Binding Protein beta Subunit/ | 12404 | |
| 21 | | cortisol.mp. | 54671 | |
| 22 | | "S100 beta".mp. | 251 | |
| 23 | | "TNF alpha".mp. or Tumor Necrosis Factor-alpha/ | 159040 | |
| 24 | | IGF-1.mp. or Insulin-Like Growth Factor I/ | 37447 | |
| 25 | | "apolioprotein E4".mp. or Apolipoproteins E/ | 16307 | |
| 26 | | "C reactive protein".mp. or C-Reactive Protein/ | 64777 | |
| 27 | | C-Reactive Protein/ or CRP.mp. | 57755 | |
| 28 | | Dopamine/ or dopamine.mp. | 146886 | |
| 29 | | neurotransmitter*.mp. | 86313 | |
| 30 | | 5 or 6 or 7 or 8 or 9 or 10 or 11 or 12 or 13 or 14 or 15 or 16 or 17 or 18 or 19 or 20 or 21 | 1146997 | |
| 31 | | 4 and 5 and 12 and 30 | 120 | |
| 32 | | limit 31 to (yr="1980 -Current" and english and humans) | 82 | |
| **MEDLINE: Cancer pain** | | | | |
| [**#**](http://ovidsp.tx.ovid.com.ezproxy.lib.uts.edu.au/sp-3.18.0b/ovidweb.cgi?&S=LAIPFPPIBODDMAHBNCJKJELBHKLIAA00&Sort+Sets=descending) | | **Searches** | **Results** | |
| 1 | | “cancer pain”.mp. or Cancer Pain/ | 6674 | |
| 2 | | (cancer adj5 pain).mp. | 11491 | |
| 3 | | 1 or 2 | 11491 | |
| 4 | | cancer.mp. or Neoplasms/ | 2831489 | |
| 5 | | Advanced.mp. | 381443 | |
| 6 | | metastasis.mp. or Neoplasm Metastasis/ | 325957 | |
| 7 | | "end stage".mp. | 63359 | |
| 8 | | "late stage".mp. | 19595 | |
| 9 | | "stage 4".mp. | 5379 | |
| 10 | | "stage four".mp. | 258 | |
| 11 | | 6 or 7 or 8 or 9 or 10 or 11 | 853407 | |
| 12 | | Biomarkers/ or biomarker*.mp. | 330208 | |
| 13 | | Cytokines/ or cytokine*.mp. | 533139 | |
| 14 | | tryptophan.mp. or Tryptophan/ | 68521 | |
| 15 | | melatonin.mp. or Melatonin/ | 32995 | |
| 16 | | serotonin.mp. or Serotonin/ | 203499 | |
| 17 | | chemokine*.mp. | 117568 | |
| 18 | | interleukin.mp. or Interleukins/ | 555852 | |
| 19 | | S100 Proteins/ or S100b.mp. or S100 Calcium Binding Protein beta Subunit/ | 24409 | |
| 20 | | cortisol.mp. | 67889 | |
| 21 | | "S100 beta".mp. | 175 | |
| 22 | | "TNF alpha".mp. or Tumor Necrosis Factor-alpha/ | 233733 | |
| 23 | | IGF-1.mp. or Insulin-Like Growth Factor I/ | 52614 | |
| 24 | | "apolioprotein E4".mp. or Apolipoproteins E/ | 23229 | |
| 25 | | "C reactive protein".mp. or C-Reactive Protein/ | 137997 | |
| 26 | | C-Reactive Protein/ or CRP.mp. | 156134 | |
| 27 | | Dopamine/ or dopamine.mp. | 203307 | |
| 28 | | neurotransmitter*.mp. | 100938 | |
| 29 | | 5 or 6 or 7 or 8 or 9 or 10 or 11 or 12 or 13 or 14 or 15 or 16 or 17 or 18 or 19 or 20 or 21 | 1743254 | |
| 30 | | 3 and 4 and 11 and 29 | 409 | |
| 31 | | limit 30 to (yr="1980 -Current" and english and humans) | 196 | |
| **MEDLINE- Fatigue** | | | | |
| [**#**](http://ovidsp.tx.ovid.com.ezproxy.lib.uts.edu.au/sp-3.18.0b/ovidweb.cgi?&S=LAIPFPPIBODDMAHBNCJKJELBHKLIAA00&Sort+Sets=descending) | | **Searches** | **Results** | |
| 1 | | "cancer fatigue".mp. or cancer fatigue/ | 147 | |
| 2 | | (cancer adj5 fatigue).mp. | 2262 | |
| 3 | | 1 or 2 | 2262 | |
| 4 | | cancer.mp. or Neoplasms/ | 2831489 | |
| 5 | | Advanced.mp. | 381443 | |
| 6 | | metastasis.mp. or Neoplasm Metastasis/ | 325957 | |
| 7 | | "end stage".mp. | 63359 | |
| 8 | | "late stage".mp. | 19595 | |
| 9 | | "stage 4".mp. | 5379 | |
| 10 | | "stage four".mp. | 258 | |
| 11 | | 5 or 6 or 7 or 8 or 9 or 10 | 853407 | |
| 12 | | Biomarkers/ or biomarker*.mp. | 330208 | |
| 13 | | Cytokines/ or cytokine*.mp. | 533139 | |
| 14 | | tryptophan.mp. or Tryptophan/ | 68521 | |
| 15 | | melatonin.mp. or Melatonin/ | 32995 | |
| 16 | | serotonin.mp. or Serotonin/ | 203499 | |
| 17 | | chemokine*.mp. | 117568 | |
| 18 | | interleukin.mp. or Interleukins/ | 555852 | |
| 19 | | S100 Proteins/ or S100b.mp. or S100 Calcium Binding Protein beta Subunit/ | 24409 | |
| 20 | | cortisol.mp. | 67889 | |
| 21 | | "S100 beta".mp. | 175 | |
| 22 | | "TNF alpha".mp. or Tumor Necrosis Factor-alpha/ | 233733 | |
| 23 | | IGF-1.mp. or Insulin-Like Growth Factor I/ | 52614 | |
| 24 | | "apolioprotein E4".mp. or Apolipoproteins E/ | 23229 | |
| 25 | | "C reactive protein".mp. or C-Reactive Protein/ | 137997 | |
| 26 | | C-Reactive Protein/ or CRP.mp. | 156134 | |
| 27 | | Dopamine/ or dopamine.mp. | 203307 | |
| 28 | | neurotransmitter*.mp. | 100938 | |
| 29 | | 6 or 7 or 8 or 9 or 10 or 11 or 12 or 13 or 14 or 15 or 16 or 17 or 18 or 19 or 20 or 21 or 22 | 1572684 | |
| 30 | | 3 and 4 and 11 and 29 | 267 | |
| 31 | | limit 37 to (yr="1980 -Current" and english and humans) | 207 | |
| **MEDLINE- Sickness behaviour search** | | | | |
| [**#**](http://ovidsp.tx.ovid.com.ezproxy.lib.uts.edu.au/sp-3.18.0b/ovidweb.cgi?&S=LAIPFPPIBODDMAHBNCJKJELBHKLIAA00&Sort+Sets=descending) | | **Searches** | **Results** | |
| 1 | | "sickness behavior".mp. | 571 | |
| 2 | | "sickness behaviour".mp. | 179 | |
| 3 | | 1 or 2 | 748 | |
| 4 | | cancer.mp. or Neoplasms/ | 2831489 | |
| 5 | | Biomarkers/ or biomarker*.mp. | 330208 | |
| 6 | | Advanced.mp. | 381443 | |
| 7 | | metastasis.mp. or Neoplasm Metastasis/ | 325957 | |
| 8 | | "end stage".mp. | 63359 | |
| 9 | | "late stage".mp. | 19595 | |
| 10 | | "stage 4".mp. | 5379 | |
| 11 | | "stage four".mp. | 258 | |
| 12 | | 6 or 7 or 8 or 9 or 10 or 11 | 853407 | |
| 13 | | Cytokines/ or cytokine*.mp. | 533139 | |
| 14 | | tryptophan.mp. or Tryptophan/ | 68521 | |
| 15 | | melatonin.mp. or Melatonin/ | 32995 | |
| 16 | | serotonin.mp. or Serotonin/ | 203499 | |
| 17 | | chemokine*.mp. | 117568 | |
| 18 | | interleukin.mp. or Interleukins/ | 555852 | |
| 19 | | S100 Proteins/ or S100b.mp. or S100 Calcium Binding Protein beta Subunit/ | 24409 | |
| 20 | | cortisol.mp. | 67889 | |
| 21 | | "S100 beta".mp. | 175 | |
| 22 | | "TNF alpha".mp. or Tumor Necrosis Factor-alpha/ | 233733 | |
| 23 | | IGF-1.mp. or Insulin-Like Growth Factor I/ | 52614 | |
| 24 | | "apolioprotein E4".mp. or Apolipoproteins E/ | 23229 | |
| 25 | | "C reactive protein".mp. or C-Reactive Protein/ | 137997 | |
| 26 | | C-Reactive Protein/ or CRP.mp. | 156134 | |
| 27 | | Dopamine/ or dopamine.mp. | 203307 | |
| 28 | | neurotransmitter*.mp. | 100938 | |
| 29 | | 6 or 7 or 8 or 9 or 10 or 11 or 12 or 13 or 14 or 15 or 16 or 17 or 18 or 19 or 20 or 21 or 22 | 1572684 | |
| 30 | | 3 and 4 and 12 and 29 | 267 | |
| 31 | | limit 27 to (yr="1980 -Current" and english and humans) | 207 | |
