# Supplementary material for: A systematic review of the overlap of fluid biomarkers in delirium and advanced cancer-related syndromes
Source: BMC Psychiatry. 2020 Apr 22;20:182. doi: 10.1186/s12888-020-02584-2 (PMC7178636; doi:10.1186/s12888-020-02584-2)
Supplement: Supplementary file 2 — Additional file 2:. Participant characteristics- delirium studies Characteristics of participants in the included delirium studies. [file 12888_2020_2584_MOESM2_ESM.docx]

## Additional file 2: Participant characteristics- delirium studies

| **Author and year** | **Country** | **Setting** | **Aims** | **Participants** | | |  |
| --- | --- | --- | --- | --- | --- | --- | --- |
|  |  |  |  | **N** | **Male, n (%)** | **Mean age; SD; (range)** | **Comorbidities** |
| Egberts *et al.* (2017) | The Netherlands | NR | To compare mean NLR levels of patients with and without delirium who were acutely admitted to a geriatric ward. | Total participants (n=86); with delirium (n=13); no delirium (n=73) | In the delirium group: 4 (30.8%); in the no delirium group: 28 (38.4%) | In the delirium group: 81.2 ± 6.6; in the no delirium group: 79.9 ± 6.5 (range NR) | NR |
| Kozak *et al.* (2017) | Turkey | Non-intensive stroke unit | To investigate whether the occurrence of delirium in patients with acute ischemic stroke (AIS) is associated with serum TNF-alpha, IL-1b, BDNF and NSE on admission. | Total participants (n=60); with ischemic stroke and delirium (n=11); with acute ischemic stroke but no delirium (n=49) | 29 (48.3%) | 66.15 ± 12.53 (range 31-89) | Cardiovascular  Diabetes |
| Tomasi *et al.* (2017) | Brazil | Respiratory Care Unit | Hypothesis: In non-severe septic patients, blood biomarkers of inflammation, endothelial activation, coagulation, and brain function would be different when compared to patients with and without brain dysfunction. | Total participants (n=38); with CAP-induced sepsis (n=20); patients with sepsis acquired encephalopathy (n=10); non-sepsis patients with delirium (n=8) | Total cohort: 19 (50%); in patients with delirium: 6 (75%) | Median age of total cohort: 60 (29-88); median age of delirium patients: 57 (38-88) | Cardiovascular  Respiratory  Diabetes |
| Vasunilashorn *et al.* (2017) | USA | Two academic medical centers | To examine associations between CRP measured preoperatively and on postoperative day 2 and delirium incidence, duration, and feature severity. | Total participants (n=560); with delirium (n=134); no delirium (n=426) | In the delirium group: 53 (39.5%); in the no delirium group: 181 (42.4%) | In the delirium group: 77.5 ± 5.0; in the no delirium group: 76.4 ± 5.2 (range NR) | Cardiovascular  Connective tissue disease |
| Chu *et al.* (2016) | China | The orthopaedic ward of a medical centre | To investigate the association between IGF-1 levels and the incidence of delirium in a homogeneous and well-defined population. | Total participants (n=103); with delirium (n=23); no delirium (n=80) | 76 (64.4%) | 81.74 ± 3.98 (range NR) | NR |
| Dillon *et al.* (2016) | USA | University teaching hospital | To identify the top candidate protein marking for delirium using plasma obtained at 4 serial time points from older patients undergoing major non-cardiac surgery. | Total participants (n=566); in the pooled cohort (n=150; with delirium (n=150); no delirium (n=150)) | In the pooled cohort: with delirium: 75 (50%); in the no delirium group: 75 (50%) | In the pooled cohort: delirium group: 77.6 ± 4.7; in the no delirium group: 77.2 ± 4.5 (range NR) | Vascular |
| Guo *et al.* (2016) | China | Department of Anaesthesiology and Critical Care Medicine | To investigate the prevalence and perioperative risk factors of POD, including medical history, comorbidities and clinical laboratory data, in elderly patients after THA for hip fracture. | Total participants (n=572); with POD (n=120); no POD (n=452) | In the delirium group: 36 (30%); in the no delirium group: 170 (37.6%) | Median age in the delirium group: 82 (76-86); in the no delirium group: 76 (72-80) | Cardiovascular  Neurological |
| Karlicic *et al.* (2016) | Serbia | Psychiatric ICU | To examine the relation between the markers of inflammation and lethal outcome in patients diagnosed with delirium and hospitalized in the intensive psychiatric care unit. | Total participants (n=120); delirious patients who survived (n=80); Delirious patients who died (n=40) | In the delirium group who survived: 68 (85%); in the deceased group: 29 (72.5%) | In delirium patients who survived: 46.8 ± 1.6; in the deceased group: 57.3 ± 13.2 (range NR) | Respiratory  Urinary |
| Neerland *et al.* (2016) | UK and Norway | University hospital | To examine whether delirium in individuals with hip fracture is associated with high CRP, IL-6, and sIL-6R levels in the CSF. | Total participants (n=149); with delirium (n=71); no delirium (n=78) | In the delirium group: 21 (29.5%); in the no delirium group: 16 (20.5%) | Median age in the delirium group: 85 (IQR 80-89); in the no delirium group: 83 (IQR 71-88) | NR |
| Shen *et al.* (2016) | China | General surgery | To investigate potential serum predictive factors including IGF-1 for POD in elderly patients after open abdominal surgery | Total participants (n=140); with POD (n=36); no POD (n=104) | In the delirium group: 17 (47.2%); in the no delirium group: 43 (41.3%) | In the delirium group: 73.8 ± 5.9; in the no delirium group: 68.8 ± 7.0 (range NR) | Cardiovascular  Diabetes  Obstructive sleep apnea |
| Sun *et al.*  (2016) | China | NR | To elucidate the pathogenesis of POD by observing the kinetics of inflammation, stress, and dementia markers in elderly oral cancer patients with POD. | Total participants (n=112); with POD (n=56); no POD (n=56) | In the delirium group: 27 (48.2%); in the no delirium group: 23 (41%) | In the delirium group: 73.2 ± 6.5; in the no delirium group: 72.7 ± 8.3 (range NR) | NR |
| Yen *et al.*  (2016) | USA | University Medical Center | To assess preoperative serum IGF-I levels as a predictor of incident delirium in non-demented elderly elective knee arthroplasty patients. | Total participants (n=98); with delirium (n=22); no delirium (n=76) | In the delirium group: 9 (40.9%); in the no delirium group: 38 (50%) | In the delirium group: 72.5 ± 4.4; in the no delirium group: 73.7 ± 5.2 | Dementia  Cardiovascular  Diabetes  Obstructive sleep apnea  Benign prostatic hypertrophy  Incontinence  Digestive |
| Avila-Funes *et al.*  (2015) | Mexico | Tertiary care hospital | To investigate the association between serum E2 levels and incidence of delirium in a sample of hospitalized elderly women. | Total participants (n=141); with delirium (n=23); no delirium (n=118) | 0%- all women | 77.8 ± 5.6 (range NR) | Dementia  Cardiovascular  Diabetes  Cancer |
| Brum *et al.* (2015) | Brazil | Hospital cancer center | To evaluate the role of BDNF and TNF-a serum levels as disease markers of delirium in oncology inpatients. | Total participants (n=70); oncology inpatients with delirium (n=17); oncology patients without delirium (n=28) and non-oncology healthy controls (n=25) | In oncology inpatients with delirium: 10 (26%); oncology patients without delirium: 13 (34%) and non-oncology healthy controls: 15 (40%) | 65.19 ± 8.29 (range 41-89) | NR |
| Egberts *et al.* (2015) | The Netherlands | Internal Medicine and Geriatrics | To investigate the levels of the potential biomarkers neopterin, IL-6 and IGF-1 in elderly patients with and without a delirium. | Total participants (n=86); with delirium (n=23); no delirium (n=63) | In the delirium group: 10 (43.5%); in the no delirium group: 30 (47.6%) | Median age in the delirium group: 87.0 (84-88); in the no delirium group: 81.0 (75-85) | NR |
| Foroughan *et al.*  (2015) | Iran | General hospital-unspecified | To investigate the occurrence of delirium and identify the associated risk factors in a sample of hospitalized elderly in Southwestern Iran. | Total participants (n=200); with delirium (n=44); no delirium (n=156) | In the delirium group: 28 (42.4%); in the no delirium group: 38 (57.6%) | In the delirium group: 78.5 ± 8.2; in the no delirium group: 70.7 ± 6 (range NR) | Dementia  Cardiovascular  Diabetes  Cancer |
| Skrede *et al.*  (2015) | Norway | University teaching hospital | To investigate the relationship between MCP-1, measured in serum preoperatively and postoperatively, and the development of delirium in a population of elderly hip fracture patients. | Total participants (n=19); pre-op delirium (n=5); POD (n=9); no delirium (n=10) | 5 (26.3%) | Median age: 83 (79-91) | NR |
| Vasunilashorn *et al.*  (2015) | USA | 3 academic medical centers | To examine the relationship between 12 cytokines (measured at 4 time points) and delirium among older adults undergoing major elective surgery. | Total participants (n=566): discovery cohort (39 delirium cases) and a replication cohort (36 delirium cases); and a pooled cohort which combined discovery and replication (n=75); Patients with no delirium and no sub-syndromal delirium on any postoperative day. Discovery cohort (n=39); replication cohort (n=36); and pooled cohort (n=75) | In the discovery delirium cohort: 18 (46%) ; in the no delirium discovery cohort: 18 (46%); in the replication delirium cohort: 23 (63.8%) and in the no delirium replication cohort: 23 (63.8%) | **Discovery cohort:** with delirium: 77.3 ± 5.0; without delirium: 76.8 ± 4.7. **Replication cohort:** with delirium: 78.0 ± 4.4; without delirium: 77.6 ± 4.2 (range NR) | **Vascular** |
| Alexander *et al.*  (2014) | USA | ICU | To determine the association between inflammatory markers, APOE, APOE genotype, and the occurrence, duration, and outcome of delirium in ICU patients. | Total participants (n=77); delirium present (n=35); no delirium (n=18) | In the delirium group: 17 (48.5%); in non-delirium group: 8 (44.4%) | Delirium group: 47.2 ± 17.4; no delirium group: 46.4 ± 18.3 (range NR) | Respiratory  Acute brain dysfunction |
| Baranyi *et al.*  (2014) | Germany | Department of cardiac surgery | To investigate the impact of sIL-2R as a biomarker of delirium after cardiac surgery with CPB. | Total participants(n=34); POD (n=11); no delirium (n=23) | 22 (64.7%) | 68.2 ± 9.7 (range NR) | NR |
| Cape *et al.*  (2014) | UK and the Netherlands | Two university teaching hospitals | To investigate 5 biomarkers known to be involved in the neuro-inflammatory process in rodents. | Total participants (n=43); with delirium (n=19); with no delirium (n=24) | In the delirium group: 5 (26.3%); in the no delirium group: 6 (25%) | In the delirium group: 81.3 ± 6.0; in the no delirium group: 81.3 ± 8.6 (range NR) | Dementia |
| Capri *et al.*  (2014) | Italy | General hospital- unspecified | To further investigate predictive factors of POD assessing pre-operative-inflammaging related-cytokines plasma level. | Total participants (n=74); with POD (n=37); no POD (n=37) | In the delirium group: 20 (54%); in the no delirium group: 17 (45.9%) | In the delirium group: 79.2 ± 6.7; in the no delirium group: 76.4 ± 6.7 (range NR) | NR |
| Chen *et al.* (2014) | China | General hospital-unspecified | To investigate the ability of plasma leptin level to predict delirium and also prolonged delirium in elderly patients after hip fracture surgery. | Total participants (n=372); with delirium (n=70); without delirium (n=116); healthy controls (n=186) | 47 (25.3%) | Total cohort: 76.7 ± 8.0 (range NR) | NR |
| Hatta *et al.*  (2014) | Japan | 4 university hospitals and 1 general hospital | To investigate whether a change in inflammatory status, indicated by blood natural killer (NK) cell activity, predicts delirium. | Total participants (n=29); patients developing delirium (n=9); no delirium (n=20) | In the delirium group: 5 (56%); in the no delirium group: 6 (30%) | In the delirium group: 77.2 ± 6.5; in the no delirium group: 81.5 ± 7.5 | Dementia Cardiovascular |
| Kazmierski *et al.*  (2014) | Poland | The cardiac surgical ICU | Primary: to assess whether patients with MCI referred for coronary artery bypass graft (CABG) surgery are at a greater risk of development of POD. Secondary aim: to investigate the putative associations between MCI and cortisol levels, as well as inflammatory and nutritional factors. | Total participants (n=113); with delirium (n=41); no delirium (n=72) | In the delirium group: 29 (70.7%); in the no delirium group: 61 (84.7%) | Median age in the delirium group: 68.8 (IQR 64-74); in the no delirium group: 61.5 (IQR 58-67.5) | Dementia  Cardiovascular  Diabetes  Depression |
| Ritchie *et al.* (2014) | UK | Medical Acute Admissions Unit | To describe the association between CRP and the incidence and severity of delirium in a large sample of elderly patients admitted to an acute hospital, and to determine if there was any interaction between CRP and delirium by diagnosis as a proxy for upstream etiologies. | Total participants (n=710); with delirium (n=87); no delirium (n=623) | In the delirium group: 33 (37.9%); in the no delirium group: 258 (41.4%) | In the delirium group: 86.7 ± 7.26; mean age in the no delirium group: 82.5 ± 7.29 (range in total cohort 70-101) | Cardiovascular  Musculoskeletal  Infection  Metabolic |
| Ritter *et al.*  (2014) | Brazil | ICU in University teaching hospital | To test the hypothesis that an association between systemic inflammatory mediators and the occurrence of delirium will differ between septic and non-septic patients. | Total participants (n=78); with delirium (n=31): out of the delirious cohort 18 (58%) of them had sepsis on admission; patients without delirium (n=47)- 21 (44%) of this cohort had sepsis at ICU admission | In the delirium group: 20 (64%); in the no delirium group: 34 (72%) | Median age in the delirium group: 56 (43-75); in the no delirium group: 57 (42-66) | Sepsis |
| Zhang *et al.* (2014) | China | ICU- teaching hospital | To examine CRP on ICU entry was associated with subsequent development of delirium. | Total participants (n=223); with delirium (n=54); no delirium (n=169) | In the delirium group: 37 (68.5%); in the no delirium group: 104 (61.5%) | In the delirium group: 64.5± 18.1; in no delirium group: 54.9 ± 16.3 (range NR) | NR |
| Cerejeira *et al.*  (2013) | Portugal | Orthopaedics | To determine the response of plasma cortisol and IGF-1 following surgical trauma, and their relationship with the innate immune response and POD. | Total participants (n=101); with delirium (n=37); no delirium (n=64) | In the delirium group: 15 (40.5%); in the no delirium group: 35 (54.7%) | In the delirium group: 73.65 ± 5.87 (64-89) ; in the no delirium group: 72.69 ± 6.53 (60-87) | NR |
| Colkesen *et al.*  (2013) | Turkey | ICU | To examine the association between serum cortisol levels and occurrence of delirium after ACS. | Total (n=52); with delirium (n=25); no delirium (n=27) | In the delirium group: 13 (52%); in the no delirium group: 15 (55%) | In the delirium group: 66 ± 6; in the no delirium group: 62 ± 9 (range NR) | Cardiovascular |
| Kazmierski *et al.* (2013) | Poland | Cardiac surgical ICU | Primary: To investigate the association between preoperative and postoperative plasma cortisol concentrations and the development of POD. Secondary: To assess whether any association between cortisol and delirium is stress related or mediated by way of MDD or cognitive impairment. | Total participants (n=113); with delirium (n=41); no delirium (n=72) | 90 (79.65%) | Median age: 64 (IQR 59-71) | Cardiovascular  Diabetes  Depression |
| Kazmierski *et al.*  (2013)b | Poland | Cardiac surgical ICU | Primary: to investigate the independent association between raised pro- inflammatory cytokine levels (IL-2 and TNF- a) and delirium diagnosed after CABG surgery. Secondary aim: to establish whether any association between raised cytokine levels and delirium is related to surgical and anesthetic procedures or mediated by pre-existing conditions associated with raised cytokine levels, such as MDD, cognitive impairment, or aging. | Total participants (n=113); with delirium (n=41); no delirium (n=72) | In the delirium group: 29 (70.7%); in the no delirium group: 61 (84.7%) | Median age in the delirium group: 68.8 (IQR 64-74); in the no delirium group: 61.5 (IQR 58-67.5) | Dementia  Depression |
| Liu *et al.* (2013) | China | University teaching hospital | To investigate the association of serum IL-6 levels with the occurrence of delirium in elderly patients after major non-cardiac surgery | Total participants (338); with delirium (n=50); no delirium (n=288) | In the delirium group: 27 (54%); in the no delirium group: 163 (56.6%) | In the delirium group: 74 ± 6; in the no delirium group: 71 ±7 (range NR) | Cardiovascular  Respiratory  Diabetes  Sepsis  Intestinal obstruction  Renal function lesion |
| Plaschke *et al.* (2013) | Germany | University teaching hospital | To explore the role of pro- and anti-inflammatory cytokines in POD in two studies. | Total participants (n=151); **Cardiac ICU**: with delirium (n=32); no delirium (n=82); **Non-cardiac ICU**: with delirium (n=17); no delirium (n=20) | In the delirium group: 21 (65.6%); no delirium group: 67 (81.7%) | **Cardiac ICU**: with delirium: 73.3 ± 6.0; without delirium: 67.3 ± 9.3. **Non-cardiac ICU**: with delirium: 64.4 ± 13.3; without delirium: 64.6 ± 10.0 | **NR** |
| Skrobik *et al.* (2013) | Canada | ICU | To compare biological and drug treatment characteristics in patients with coma and/or delirium while in the ICU. | Total participants (n=99); with delirium (n=64); with coma (n=59); no coma and no delirium (n=12) | In the delirium group: 31 (48.4%); in the coma group: 55 (55.4%); in the no coma and no delirium group: 7 (58.3%) | In the delirium group: 62.0 ± 13.9; in the coma group: 63.2 ± 14.2; in the no delirium and no coma group: 55.2 ± 15.7 (range NR) | Hepatic dysfunction  Renal dysfunction |
| Westhoff *et al.* (2013) | The Netherlands | Teaching hospital | Hypothesis: Elderly hip fracture patients with altered CNS cytokine profiles before surgery,  are specifically at risk for developing delirium postoperatively. Aim: to study the hypothesis by analysing a range of pro- and anti-inflammatory markers in CSF in elderly  patients undergoing emergency hip surgery. | Total participants (n=61); with delirium (n=23); no delirium (n=38) | In the delirium group: 7 (30.4%); in the no delirium group: 12 (31.5%) | In the delirium group: 84.6 ± 5.2; in the no delirium group: 82.9 ± 4.5 (range NR) | NR |
| Bakker *et al.*  (2012) | The Netherlands | The Department of Cardiothoracic Surgery | To identify preoperative and operative characteristics that enable the prediction of delirium after cardiac surgery in elderly patients. | Total participants (n=201); with delirium (n=63); no delirium (n=138) | In the delirium group: 37 (59%); in the non-delirium group: 84 (61%) | Delirium group: 76.7 ± 3.9; in the no delirium group: 75.9 ± 3.7 (range NR) | Cardiovascular  Diabetes |
| Baranyi *et al.*  (2012) | Germany | Department of cardiac surgery | To investigate the impact of intra- and postoperative albumin levels as a biomarker of delirium after cardiac surgery with CPB. | Total participants (n=34); POD (n=11); no delirium (n=23) | 22 (64.7%) | 68.2 ± 9.7 (range NR) | NR |
| Cerejeira *et al.*  (2012) | Portugal | General hospital orthopedic ward | To clarify whether delirium is associated with an unbalanced inflammatory response or a dysfunctional interaction between the cholinergic and immune systems. | Total participants (n=101); with delirium (n=37); no delirium (n=64) | In the delirium group: 15 (40.5%); in the no delirium group: 35 (54.7%) | 73 ± 6.3 (range 60–89) | NR |
| Girard *et al.*  (2012) | USA | General hospital-unspecified | To assess the associations between a priori-selected markers of inflammation and coagulation and delirium during critical illness. | Total participants (n=138); with delirium (n=107); no delirium (n=31) | 69 (50%) | Median age: 66 | Cardiovascular  Respiratory  Sepsis  Stroke/intracranial haemorrhage  Renal Failure |
| Osse *et al.*  (2012) | The Netherlands | University hospital | To examine the association between plasma levels of pterins and amino acids and POD. | Total participants (n=125); with delirium (n=58); no delirium (n=67) | In the delirium group: 34 (58.6%); in the no delirium group: 48 (71.6%) | In the delirium group: 76.7 ± 3.9; and in the no delirium group: 75.1 ± 3.1 (range NR) | Cardiovascular  Diabetes |
| Bisschop *et al.*  (2011) | The Netherlands | Department of Orthopedic Surgery or Traumatology | To evaluate a possible relationship between glucose, cortisol, insulin, and delirium. | Total participants (n=143); with delirium (n=70); no delirium (n=73) | In delirium group: 17 (24%); in the no delirium group: 26 (36%) | Delirium group: 85.1 ± 6.7; in the no delirium group: 82.6 ± 6.9 (range NR) | Cardiovascular Preadmission cognitive impairment  Diabetes |
| Holmes *et al.* (2011) | UK | Memory assessment services | To determine if raised serum TNF-a or IL-6 are associated with the presence of sickness behaviour symptoms, independent of the development of delirium, in a prospective cohort study of participants with AD. | Total participants with mild to severe AD (n=222); with delirium (n=197); without delirium (n=25) | 102 (34%) | 82.8 ± 0.4 | NR |
| Lee *et al.*  (2011) | Korea | Orthopaedic surgery | To identify predictive factors of delirium, including risk factors and prodromal symptoms. | Total participants (n=65); with delirium (n=18); no delirium (n=47) | In the delirium group: 8 (44.4%); in the no delirium group: 14 (29.7%) | In the delirium group: 81.7 ± 6.35 (69-94); in the no delirium group: 75.0 ± 7.83 (65-90) | NR |
| McGrane *et al.* (2011) | USA | Two tertiary care centers | To test the hypothesis that systemic inflammation, as measured by the inflammatory biomarkers procalcitonin and CRP, is associated with a longer duration of brain dysfunction in mechanically ventilated patients. | Total participants (n=87) | 44 (50%) | Median age: 60 (IQR 49-66) | Sepsis |
| Morandi *et al.*  (2011) | USA | General hospital-unspecified | To prospectively test the hypothesis that low IGF-1 concentrations early during critical illness would be associated with delirium in mechanically ventilated medical ICU patients. | Total mechanically ventilated medical ICU patients in entire sample (n=110); patients included in primary analysis=62) | In the entire sample: 57 (52%); in the patients included in the primary analysis: 35 (57%) | Median age in the entire sample: 65 (IQR 52-74); in the patients included in the primary analysis: 66 (53-76) | Cardiovascular  Respiratory  Sepsis |
| Van der Boogaard *et al.* (2011)a | The Netherlands | ICU | To examine plasma biomarkers in delirious and non-delirious patients and the role of these biomarkers on long-term cognitive function. | Total participants (n=100); with delirium (n=50); no delirium (n=50) | In the delirium group: 27 (46%); in the no delirium group: 26 (40%) | In the delirium group: 72 (95% CI 38-86); in the no delirium group: 68 (95% CI 31-84) | NR |
| Van der Boogaard *et al.*  (2011)b | The Netherlands | ICU | To explore whether biomarkers associated with delirium could be detected in urinary protein profiles of hyperactive delirium compared to matched non-delirium ICU- patients. | Total participants (n=20); with hyperactive delirium (n=10); no delirium (n=10) | In the delirium group: 7 (70%); in the no delirium group: 6 (60%) | Median age in the delirium group: 75 (IQR 70-78); in the no delirium group: 75 (IQR 68-78) | NR |
| Burkhart *et al.*  (2010) | Switzerland | University teaching hospital | To identify modifiable risk factors associated with the development of POD in elderly patients after elective cardiac surgery in order to be able to design follow-up studies aimed at the prevention of delirium by optimizing perioperative management. | Total participants (n=113); with delirium (n=35); without delirium (n=78) | 77 (68%) | 74.3 ± 5.51 (range NR) | Cardiovascular  Diabetes  Renal insufficiency |
| Mu *et al.*  (2010) | China | General hospital-unspecified | To examine the association between serum cortisol level and occurrence of early POD in patients undergoing CABG surgery. | Total participants (n=243); with delirium (n=123); no delirium (n=120) | In the delirium group: 101 (82.1%); in the no delirium group: 99 (82.5%) | In the delirium group: 63.6 ± 7.7; in the no delirium group: 58.3 ± 8.0 (range NR) | Cardiovascular  Respiratory  Sepsis |
| Pearson *et al.* (2010) | UK | NR | To test the hypothesis that delirium is associated with higher CSF and plasma cortisol levels in older patients with acute hip fracture. | Total participants (n=20); with delirium (n=7); no delirium (n=13) | In the delirium group: 1 (14.2%); in the no delirium group: 4 (30.7%) | In the delirium group: 81.4 ± 7.2; in the no delirium group: 80.5 ± 8.7; (range of total cohort 62-93) | Cardiovascular  Respiratory  Diabetes  Rheumatoid arthritis |
| Plaschke *et al.* (2010)^[[1]](#footnote-1)^ | Germany | Cardiac surgical ICU | To analyse whether the BIS, cortisol, and IL-6 were different in delirious patients as compared to non-delirious ones after cardiac surgery. | Total participants (n=114); with delirium (n=32); no delirium (n=82) | 89 (78%) | In the delirium group: 73.3 ± 6.0; in the no delirium group: 67.3 ± 9.3 (range NR) | Cardiovascular  Diabetes |
| Tsruta *et al.* (2010) | Japan | University teaching hospital- Advanced Medical Emergency & Critical Care Center | To investigate the prevalence and associated factors of delirium in critically ill patients during an ICU stay. | Total participants (n=103); with delirium (n=21); no delirium (n=82) | In the delirium group: 13 (62%); in the no delirium group: 51 (62%) | In the delirium group: 70 ± 17; in the no delirium group: 64 ± 19 (range NR) | Cardiovascular  Respiratory  Digestive  Trauma/burns  Acute poisoning |
| Van Munster *et al.* (2010) | The Netherlands | Department of Orthopedic Surgery /Traumatology of an Academic Medical Centre | To investigate the levels of cortisol in a large sample and compare the levels cortisol, IL-6 and IL-8 and S100B in one study among elderly patients with hip fracture with and without delirium. | Total participants (n=120); with delirium (n=62); without delirium (n=58) | In the delirium group: 16 (26%); in the no delirium group: 23 (40%) | In the delirium group: 84.8 ± 6.9; in the no delirium group: 82.9 ± 7.9 (range NR) | NR |
| Adamis *et al.* (2009) | UK | Elderly care unit | To investigate the relationship of serum cytokines, IGF-I, severity of illness, cognition, possession of APOE epsilon 4 genotype, gender and age on (i) the presence of delirium and (ii) on its severity. | Total participants(n=67); with delirium (n=28); no delirium (n=39) | 19 (28.3%) | 84.2 ± 6.3 (70–94) | Dementia  Cardiovascular  Respiratory  Urinary tract infection  Falls  Cellulitis |
| Van Munster *et al.* (2009) | The Netherlands | Academic Medical Centre | (1) to compare changes before and after surgery of S100B and NSE levels in serum in patients with and without POD, and to investigate the difference in serum levels before, during and after delirium; (2) to study the serum levels of S100B and NSE in different subtypes of delirium. | Total participants (n=120); patients with delirium (n=62); no delirium (n=58) | In the delirium group: 16 (26%); in the no delirium group: 23 (40%) | In the delirium group: 84.8 ± 6.9; in the no delirium group: 82.9 ± 7.0 | NR |
| Lemstra *et al.* (2008) | The Netherlands | Teaching hospital | To investigate the association of cytokine levels and incident delirium in a homogeneous and well-defined population. | Total participants (n=68); with POD (n=18); no POD (n=50) | In the delirium group: 8 (44.4%); in the no delirium group: 13 (26%) | NI | Neurological  Respiratory  Endocrine  Psychiatric |
| Pfister *et al.* (2008) | Switzerland | ICU | To test the hypothesis that cerebral perfusion and selected serum markers of inflammation and delirium differ in septic patients with and without sepsis-associated delirium. | Total participants (n=16); with sepsis-associated delirium (n=12); Patients with no sepsis-related delirium (n=4) | 14 (62%) | Median age: 74.5 (18-90) | NR |
| Rudolph *et al.* (2008) | USA | An academic medical center | To determine if a difference exists in the postoperative pattern of change in a priori determined classes of inflammatory markers in matched patients with and without delirium after cardiac surgery. | Total participants (n=42); with delirium (n=12); no delirium (n=30) | In the delirium group: 11 (92%); in the no delirium group: 9 (75%) | In the delirium group: 74.7 ± 7.0; in the no delirium group: 73.9 ± 8.4 (range NR) | Cardiovascular  Diabetes |
| Van Munster *et al.* (2008) | The Netherlands | Department of Orthopedic Surgery /Traumatology of an Academic Medical Centre | To compare the time-course of cytokine expression in elderly patients with hip fracture with and without POD and investigate the possible associations between cytokines and different subtypes of delirium. | Total participants (n=98); with delirium (n=50); no delirium (n=48) | In the delirium group: 13 (26%); in the no delirium group: 18 (37.5%) | In the delirium group: 84.6 ± 7.1; in the no delirium group: 83.2 ± 6.7 (range NR) | NR |
| Adamis *et al.* (2007) | UK | Elderly care unit | To investigate the relationship between physical illness severity and delirium, and the relationship between putative marker of predisposition and perpetuation (APOE epsilon4 allele APOE4, CRP and cytokines) of delirium. | Total participants n=164; consented for laboratory tests (n=116); delirium present on first assessment (n=42); subsequently (n=5); no delirium (n=117) | 54 (32.9%) | 84.6 ± 6.57 (70-104) | Dementia |
| de Rooij *et al.*  (2007) | The Netherlands | General hospital-unspecified | To compare the expression patterns of pro- and anti-inflammatory cytokines in patients with and without delirium. | Total participants (n=185); with delirium (n=64); no delirium (n=121) | In the delirium group: 22 (34%); in the no delirium group: 54 (45%) | In the delirium group: 81.2± 7.1; in the no delirium group: 77.3 ± 8.0 (range NR) | Cardiovascular  Cancer  Infectious disease  Water/electrolyte disturbances |
| Plaschke *et al.* (2007) | Germany | ICU | To examine whether measurement of SAA level is a reliable indicator of delirium in ICU patients, and whether there is a significant relationship between SAA and quantitative EEG data and the clinical diagnosis of delirium using the CAM-ICU. | Total participants (n=37); with delirium (n=17); without delirium (n=20) | In the delirium group: 12 (70.5%); in the no delirium group: 15 (75%) | In the delirium group: 62.7 ± 13.2; in the no delirium group: 64.5 ± 9.9 | Cardiovascular  Digestive  Pancreas/liver failure |
| White *et al.* (2005) | UK | Emergency medical admissions | To investigate the activities of plasma esterases (drug metabolising enzymes) in delirium. | Total participants (n=283); with delirium (n=105); no delirium (n=178) | 177 (41.3%) | 82.4 ± 0.3 | Dementia |
| Wilson *et al.* (2005) | UK | Acute medical ward | To determine if low base line IGF-1 levels is a risk factor for incident delirium in patients aged 75 and over admitted to an acute medical ward. | Total participants (n=100); with delirium (n=12); no delirium (n=88) | 31 (31%) | 84.5 ± 4.2 (range NR) | Depression |
| Beloosesky *et al.* (2004) | Israel | NR | To determine the kinetics of CRP, fibrinogen and ESR in aged patients with hip fractures, over a month after surgery and to examine the relationship of these parameters to cognition, post-operative complications, functional level after 1 month and 6-month post-operative mortality. | Total participants (n=32); delirium present (n=10); no delirium (n=22) | 9 (28.1%) | 85.1 ± 4.8 years (77–97) | Cardiovascular  Respiratory  Diabetes  Digestive  Urinary |
| Robertsson *et al.* (2001) | Sweden | A hospital neuropsychiatric diagnostic unit | To determine activity in the HPA in demented patients by measuring their basal serum cortisol levels and performing DST and to ascertain whether the stress regulating system was more disturbed in the patients with delirium than in those without delirium. | Total participants (n=172); with delirium (n=67); no delirium (n=105) | NR | 69.8 ± 6.9 (52-79) | Dementia |
| Van der Mast  *et al.* (2000) | The Netherlands | Thorax centre of a University Hospital | To examine the interrelationships between the plasma levels of amino acids, physical condition, and POD in patients undergoing elective cardiac surgery. | Total participants (n=296) ; with POD (n=40); no POD (n=256) | 192 (65%) | 63 ± 11 (range 26–83) | Cardiovascular |
| Van der Mast *et al.*  (1999) | The Netherlands | NR | To investigate the incidence of delirium after various types of cardiac surgery, and associated preoperative predictors. | Total participants (n=296); with delirium (n=40); no delirium (n=256) | 192 (65%) | 63 ± 11 (26–83) | NR |
| Gustafson *et al.*  (1993) | Finland | Stroke unit | To investigate the relationships between the activity of HPA axis and ACS in patients with acute supratenrorial ischemic stroke | Total participants (n=155); with a supratentorial cerebral infarction (n=83); healthy control group (n=72) | Of the stroke patients: 52 (63%); healthy control NR | Stroke patients: 74.8 ± 8 (44-89); healthy controls: 69.2 ± 10 | Dementia  Cardiovascular  Diabetes  Stroke |
| McIntosh *et al.*  (1985) | USA | The Boston Veterans Administration Hospital | To measure the levels of plasma cortisol and B-endorphin in patients who underwent elective surgery in order to determine whether there is a relation between circulating levels of these hormones and POD. | Total participants (n=7); with delirium (n=3); no delirium (n=4) | 7 (100%) | Mean age NR; (42-65) | NR |

##

**Abbreviations::** ACS: Acute confusional state; AD: Alzheimer’s disease; APOE: Apolipoprotein E; BDNF: Brain-derived neurotrophic factor; BIS: Bispectral Index; CNS: Central nervous system; CPB: Cardiopulmonary bypass; CRP: C-reactive protein; CSF: Cerebrospinal fluid; DST: Dexamethasone suppression test; E2: Estradiol; EEG: Electroencephalography; HPA: Hypothalamic- Pituitary-Adrenal axis; ICU: Intensive care unit; IGF: Insulin-like growth factor; IL-: Interleukin; IQR: Interquartile range; MCI: Mild cognitive impairment; MCP: Monocyte chemoattractant protein; MDD: Major depressive disorder; NLR: Neutrophil/Lympthocyte ratio; NR: Not reported; NSE: Neuron-Specific Enolase; POD: Post-operative delirium; S100b: Calcium binding protein B; SAA: Serum anticholinergic activity; sIL-: Soluble interkeukin; THA: Total hip arthroplasty; TNF-a: Tumor necrosis factor- alpha

1. Same cohort as Plaschke, 2007 [↑](#footnote-ref-1)
