# Supplementary material for: A systematic review of the overlap of fluid biomarkers in delirium and advanced cancer-related syndromes
Source: BMC Psychiatry. 2020 Apr 22;20:182. doi: 10.1186/s12888-020-02584-2 (PMC7178636; doi:10.1186/s12888-020-02584-2)
Supplement: Supplementary file 3 — Additional file 3:. Participant characteristics- cancer studies Characteristics of participants in the included cancer studies. [file 12888_2020_2584_MOESM3_ESM.docx]

## Additional file 3: Participant characteristics- cancer studies

| **Author and year** | **Country** | **Setting** | **Aims** | **Participants** | | | | | |
| --- | --- | --- | --- | --- | --- | --- | --- | --- | --- |
|  |  |  |  | **N** | **Male, n (%)** | **Mean age; SD; (range)** | **Type of cancer** | **% advanced cancer** | **Cancer stage** |
| Amano *et al*. (2017) ^[[1]](#footnote-1)^ | Japan | Palliative care | To investigate the association between CRP level, symptoms, and ADL in advanced cancer patients receiving palliative care. | Total participants with advanced cancer (n=1702) | 1003 (58.9%) | 68.4 ± 12.7 (range NR) | Mixed | 100% | NR |
| Fogelman *et al*. (2017) | USA | NR | To identify which symptoms or serum markers can best predict weight loss in patients with locally advanced or metastatic pancreatic cancer. | Total participants with baseline serum for analysis (n=69); with cancer (n=63); healthy controls with no cancer diagnosis (n=6) | In the weight loss group: 14 (32.6%); in the no weight loss group: 29 (67.4%) | In the weight loss group (at diagnosis): 61.5 ± 9.4 (45.9-78; in the no-weight loss group (at diagnosis): 62.9 ± 11.4 (36-86) | Pancreatic cancer | 100% | NR |
| Luo *et al*. (2017) | Korea | NR | Aim of cohort study: To evaluate the effect of elevated plasma fibrinogen levels for predicting the prognosis of advanced-stage EOC compared with serum CA-125 levels and systemic inflammatory biomarkers, such as NLR and PLR. | Total participants with advanced cancer (n=217) | 0 (0%) | Median age: 54.4 (range 25–84) | Ovarian | 100% | 3 (1.4%) stage IIIA, 15 (6.9%) stage IIIB, 149 (68.7%) stage IIIC, and 50 (23%) stage IV. |
| Paulsen *et al*. (2017) | Norway | NR | Primary aim: To examine the relationship between inflammatory biomarkers (cytokines and markers of the inflammatory response) and PROMs of pain, appetite and fatigue in patients with advanced cancer receiving opioids. Secondary aim: To explore whether levels of baseline inflammatory biomarkers were associated with changes in pain, appetite and fatigue following treatment with corticosteroids. | Total participants with cancer (n=49) | 25 (51%) | 63.9 (CI 61.2-66.8) | Mixed | 96% | NR |
| Amano *et al*. (2016) | Japan | Palliative care | To investigate the clinical implications of CRP as a prognostic marker in advanced cancer patients in palliative settings. | Total participants with advanced cancer (n=1511) | 895 (59%) | Mean age in group one (CRP<1): 68.8 ± 13.4 ; mean age in group two (CRP ≤ 1): 69.1 ± 12.1; mean age in group 3 (CRP ≤ 5): 68.4 ± 12.6 and mean age in group 4 (CRP ≤ 10)= 66.3 ± 13.4 (range NR) | Mixed | 100% | NR |
| Bye *et al*. (2016) | Norway | University Hospital- unspecified | To study changes in inflammatory biomarkers and energy intake in an unselected cohort of pancreatic cancer patients with and without cachexia as they approached the terminal stage of disease. | Total participants (n=60); with cancer (n=20); healthy controls (n=40) | 15 (75%) | Median age: 67.5 (range 35-79) | Pancreatic | 100% | 6 (30 %) patients had locally advanced cancer, 13 (65 %) had metastatic disease and one (5%) had recurrent disease after an earlier pancreatic resection. |
| Mitsunga *et al*. (2016) | Japan | Cancer centre | To establish a classification using CRP level to stratify the aggressiveness of treatment-naive advanced prostate cancer in patients undergoing first-line chemotherapy. | Total participants with cancer (n=421); Retrospective cohort (n=280); prospective cohort (n=141) | In the retrospective cohort: 122 (43.6%); in the prospective cohort: 75 (53.2%) | Median age in the retrospective cohort: 63 (IQR 57-70); median age in the prospective cohort: 67 (IQR 62-74) | Pancreatic cancer | 100% | **Prospective cohort:** 21.3% stage III, 78.7% stage IV; **Retrospective cohort**: 11.8% stage III; 88.2% stage IV |
| Morgado *et al*. (2016) | Argentina | NR | To evaluate the relationship between weight loss and several parameters of skeletal muscle function. | Total participants with cancer and fatigue (n=49); Arm A: patients with ≥5% weight loss in the last 6 months (n=27); Arm B: advanced cancer patients without weight loss (n = 22) | In the weight loss group: 20 (74.1%); in the weight-stable group: 11 (50%) | Mean age in the weight loss group: 62 (39-85); in the weight-stable group: 60 (24-79) | Mixed | 100% | NR |
| Rodrigues *et al*. (2016) | Brazil | NR | To characterize the incidence of fatigue in the context of advanced cancer not influenced by cancer treatment and to assess the clinical and laboratory factors associated with this symptom. | Total participants with advanced cancer (n=51); no control | 24 (47.1%) | Median age 64 (33-85) | Mixed | 100% | NR |
| Srdic *et al*. (2016) | Croatia | University hospital- Department for Respiratory Diseases | Primary aim: To obtain prevalence of cancer cachexia and sarcopenia in patients with advanced lung cancer using criteria for definition and diagnosis. Secondary aim: To assess determinants for chemotherapy toxicity and prognostic factors for survival. | Total participants with advanced cancer (n=100); with cancer cachexia (n=69); no cachexia (n=31) | 67 (67%) | Mean age: 64 (IQR 41-87) | NSCLC | 100% | 34 % stage IIIB 66% stage IV |
| Wu *et al*. (2016) | China | Department of Colorectal Surgery | To examine the correlations of NLR and PLR with chemotherapy sensitivity and prognosis. | Total participants with advanced cancer (n=55) | 35 (64%) | 28 (51%) of patients were < 60 years old; 27 (49%) of patients were ≥ 60 years old. | Lung | 100% | 14 (35%) stage IV; 13 (30%) stage IIIB and 6 (15%) stage IIIA |
| Bilir *et al*. (2015) | Turkey | University medical oncology centre | To investigate the possible etiologic factors of cachexia | Total participants (n=80); with cancer cachexia (n=46); healthy participants with no known chronic disease or weight loss (n=34) | In the cachexia group: 36 (78.2%); in the control group: 24 (70.5%). | In the cachexia group: mean age: 60.9 ± 14; in the control group: 57.8 ± 12 (range NR) | Mixed | 100% | NR |
| Miura *et al*. (2015) | Japan | Cancer centre | To characterize IL-6 related factors in patients who were scheduled to undergo first-line chemotherapy for treatment-naïve advanced pancreatic cancer. | Total participants with advanced cancer (n=79) | 677 (58.4%) | In the <60 group: 342 participants (29.5%); in the 65-74 range: 340 participants (29.3%); in the ≥ 75 age group: 477 participants (41.2%) | Pancreatic cancer | 100% | 35.5% stage III cancer, 64.5% stage IV |
| Miura *et al*. (2015)b | Japan | Palliative care | To clarify the value of the GPS as a prognostic score in advanced cancer patients receiving palliative care services. | Total participants with advanced cancer (n=1160) | 677 (58.4%) | In the <65 group age group: 342 participants (29.5%); in the 65-74 range: 340 participants (29.3%); in the ≥ 75 age group: 477 participants (41.2%). | Mixed | 100% | NR |
| Barrera *et al*. (2014) | Mexico | University Medical Oncology Clinic | To associate the plasma levels of several cytokines with clinical characteristics and prognosis in patients with advanced NSCLC. | Total participants (n=135); with advanced cancer (n=110); healthy controls (n=25) | 47 (42.5%) | 58.5 ± 16.4 | NSCLC | 100% | 12.5% were in clinical stage IIIB, the rest were stage IV |
| Blakely *et al*. (2014) | USA | Palliative surgery | To determine if preoperative CRP is associated with patient outcomes following palliative surgery. | Total participants (n=50); patients with normal CRP (n=23); patients with elevated CRP levels (n=27) | In the normal CRP group: 11(47.8%); in the elevated CRP group: 15 (55.5%). | In the normal CRP group: mean age: 63 ± 13 (44-93); in the elevated CRP group: 63 ± 15.4 (23-88) | Mixed | 100% | NR |
| Fujiwara *et al*. (2014) | Japan | University hospital- unspecified | To investigate the difference in serum metabolite levels between pancreatic cancer patients with and without cachexia and to explore the pattern and intra-day variations in metabolite levels using metabolomics. | Total participants with advanced cancer (n=21); with cachexia (n=9); without cachexia (n=12) | In the cachexia group: 8 (8.8%); in the non-cachexia group: 8 (66.6%) | Median age in cachexia group: 66.5 (range 36-77); in the no-cachexia group: 68.5 (range 39-76) years | Pancreatic | 100% | 10 (48%) stage IVA and 11 (52%) stage IVB |
| Lindemann *et al*. (2014) | Australia | NR | To evaluate the influence of elevated CRP levels as well as hypoalbuminemia on the further survival in patients with advanced inoperable cancer affecting specifically the esophagus. | Total participants with advanced cancer (n=218) | 185 (84.9%) | 67 ± 11.84 years (21–93) | Esophageal | 100% | NR |
| Mondello *et al*. (2014) | Italy | Oncology- hospital | To investigate the role of leptin, ghrelin and obestatin as diagnostic and predictive markers of cachexia in oncologic patients. Their impact on patient survival was also evaluated. | Total participants (n=170); with advanced cancer (n=140); healthy controls (n=30) | 74 (52.8%) | Mean age in the cancer group: 61.8 ± 14.3; in the control group: 59.6 ± 12.2 (range NR) | Mixed | 100% | 25% stage III, 75% stage IV |
| Moriwaki *et al*. (2014) | Japan | NR | To evaluate the prognostic value of GPS in Biliary tract cancer patients with good ECOG PS undergoing chemotherapy. | Total participants with advanced cancer (n=62) | 33 (53%) | Median age: 68 (44-85) | Biliary tract cancer | 100% | NR |
| Szkandera *et al*. (2014) | Austria | Oncology | To validate the prognostic significance of pre-treatment plasma CRP levels on CSS in a large cohort of 474 pancreatic cancer patients. | Total participants with cancer (n=474) | 256 (54%) | Mean age at diagnosis: 64.6 ± 10.4 (range NR) | Pancreatic cancer | 77.3% | 1% stage I, 3.8% stage IIA, 17.9% stage IIB, 7% stage III, 70.3% stage IV |
| Zhang *et al*. (2014) | China | University hospital- oncology | To determine if there was a significant correlation between CRF and chemotherapy-associated adverse effects and plasma levels of TNF-α and IL-1 as well as urinary 17-HCS before and after chemotherapy | Total participants with cancer (n=200) | 118 (59%) | 64 (32%) of patients were < 40 years old; 85 (42.5%) were between 40-60 years old; and 51 (25.5%) were > 60 | Mixed | 79% | 13.5% stage II, 56.5% stage III, 22.5% stage IV |
| Jafri *et al*. (2013) | USA | NR | To see ALI at the time of diagnosis can predict survival outcomes in patients with newly diagnosed metastatic NSCLC. | Total participants with advanced cancer (n=173) | 116 (67%) | Median age: 57 (34-88) | NSCLC | 100% | All stage IV |
| Laird *et al*. (2013) | Switzerland, Germany, Denmark, UK, Iceland, Italy, Norway, and Sweden. | Multiple centres (e.g., hospital inpatients, hospital outpatients, hospices/specialist palliative care units) | To examine the relationship of pain, other key symptoms, and systemic inflammation in a large international cohort of patients with advanced cancer. | Total participants with advanced cancer (n=1466) | 739 (50%) | Median age: 62 (IQR 54-70) | Mixed | 100% | NR |
| Laird *et al*. (2013)b | Switzerland, Germany, Denmark, Australia, UK, Iceland, Austria, Italy, Norway, Sweden, and Canada. | Multiple (hospital inpatients, hospital outpatients, hospices/specialist palliative care units) | 1) to compare the prognostic value of established clinical factors with the systemic inflammation-based mGPS; 2) to assess whether performance status in combination with mGPS is more powerful than either alone; and 3) to assess both of these aspects in a test sample before validation in an independent sample. | Total participants with cancer (n=2456) | In the test sample: 931 (51%); in the validation sample: 237 (53%) | Median age in the test sample: 63 (IQR 54-71); in the validation sample: 64 (IQR 56-71) | Mixed | 100% | NR |
| Paiva *et al*. (2013) | Brazil | Palliative care | Primary aim: To evaluate the prevalence of CRF among advanced cancer patients undergoing their first consult in palliative care and to access its impact on QOL. Secondary aim: To investigate the association of CRF with known and possible predictors, as well as to determine the prognostic impact of CRF and its relationship with the inflammatory marker CRP was evaluated. | Total participants with cancer (n=223); with cancer-related fatigue (n=55); without cancer-related fatigue (n=168) | 112 (50.7%) | 60.4 ± 12.6 (21-86) | Mixed | 100% | NR |
| Suh *et al*. (2013) | Korea | 3 hospice and palliative care centre | To investigate whether plasma levels of IL-6 or TNF-α could predict survival in patients with far advanced cancer | Total participants with advanced cancer (n=98) | 52 (53.1%) | 52 (53%) of patients were ≥ 65 years old; 46 (47%) of patients were < 65 years old | Mixed | 100% | NR |
| De Raaf *et al*. (2012) | The Netherlands | Palliative care | To determine in both advanced cancer patients and cancer survivors: 1) which inflammatory markers are related to physical fatigue and mental fatigue, and 2) whether inflammatory markers that are associated with fatigue are related to each other. | Total participants (n=92); with advanced cancer (n=45); cancer survivors (n=47) | In the cancer group: 18 (40%); in the cancer survivor group: 19 (40%) | Mean age in the cancer group: 58 (22-81); in the cancer survivor group: 57 (36-77) | Mixed | 100% | NR |
| Gioulbasanis *et al*. (2012) | Greece | Oncology- university hospital | To investigate the possible association between baseline IL-8 plasma levels and nutritional status, and to evaluate the predictive and prognostic value of IL-8 in patients with NSCLC. | Total participants with cancer (n=114) | 101 (88.6%) | Median age: 67.5 ± 5.4 (range NR) | NSCLC | 100% | All stage IV |
| Gulen *et al*. (2012) | Turkey | NR (control group from Chest Diseases Outpatient Clinic) | To investigate the relationship of adipokines and systemic inflammation in weight-losing advanced-stage NSCLC patients. | Total participants (n=88); with cancer (n=63); further divided into subgroups as those with a >5% weight loss in preceding 6 months (n=33) and those who had not (n=30); healthy controls (n=25) | All male (100%) | Mean age for the cancer group: 65.63 ± 9.87 and for the control group: 63.52 ± 11.54 (range of total cohort 52-84) | NSCLC | 100% | 43% stage III and 57% stage IV |
| Heitzer *et al*. (2012) | Austria | NR | Primary aim: To identify biological, measurable biomarkers in serum correlating with pain intensity in patients with cancer.  Secondary aim: to assess cytokine serum level differences between patients and healthy controls and to evaluate possible relationships between pain entities, pain intensity, gender, location of the primary tumour, and the patients’ cytokine baseline concentrations. | Total participants (n=65); with cancer pain (n=45); healthy individuals without pain (n=20) | 17 (44.7%) | 63.1 ± 11.5 (43-89) | Mixed | 100% | NR |
| Minton *et al*. (2012) | Norway, UK, Austria, Germany, Switzerland, Italy, Canada, and Australia | Palliative care, hospices, general oncology and medical wards | To identify factors independently associated with fatigue and to determine the prevalence of severe fatigue in a diverse group of palliative care cancer patients across a variety of settings and in different countries. | Total participants with cancer in the fatigue subset analysis (n=720) | In the fatigue group: 162 (50%); 233 (56%) in the no fatigue group | Mean age in the fatigue group: 63.4; mean age in the no fatigue group: 62.5 (range NR) | Mixed | 100% | NR |
| Partridge *et al*. (2012) | UK | Palliative care | Primary aim: To examine whether mGPS is of use in cancer patients near the end of life. Secondary aim: To examine the usefulness of the mGPS at two and four weeks, which are clinically significant time points. | Total participants with biomarkers recorded (n=102); in mGPS 0 group (n=16); in mGPS 1 group: (n=20); in mGPS 2 (n=66) | In the mGPS 0 group: 8 (17.4%); in the mGPS 1 group: 9 (19.6%); in the mGPS2 group: 29 (63%). | Median age in the mGPS 0 group: 73; in the mGPS 1 group: 76; and in the mGPS 2 group: 71. | Mixed | 100% | NR |
| Pond *et al*. (2012) | Russia and USA | NR | To evaluate and compare the prognostic abilities of the aforementioned prognostic classifiers and to investigate the ability of CRP to enhance their prognostic abilities. | Total participants (n=220) | 100% | NR | Prostate | 100% | NR |
| Wang *et al*. (2012) | China | University hospital- cancer centre | To compare the prognostic value of pre-therapy CRP-based prognostic scores such as the mGPS and PI with those based on the cellular components of the systemic inflammatory response such as the NLR, PLR and PNI in patients with pancreatic cancer. | Total participants with cancer (n=177) | 120 (67.7%) | 125 patients were < 65 years old; 53 patients were ≥ 65 years old | Pancreatic cancer | 79% | 21% stage I and II, 79% stage III and IV |
| Aydin *et al*. (2011) | Turkey | Thoracic Surgery Department | To investigate the prognostic value of serum CRP, pre-albumin, and transferrin levels in patients with advanced stage esophageal cancer treated with stent placement | Total participants (n=61) | 29 (47.5%) | 63.9 ± 13.5 (range 34-94) | Esophageal cancer | 100% | NR |
| Dev *et al*. (2011) | USA | Supportive Care Clinic at University Cancer Centre | To assess the relationship between opioid use and serum cortisol and testosterone levels and explore the association of cortisol with symptoms as measured by the ESAS in patients with advanced cancer. | Total patients with advanced cancer (n=77) | 48 (62%) | Median age: 63 (51.5-69) | Mixed | 100% | NR |
| Gioulbasanis *et al*. (2011) | Greece | Oncology- university hospital | To evaluate the correlation of MNA with laboratory markers of inflammation/cachexia in patients with metastatic lung cancer. | Total participants (n=115); group A with no nutritional sufficiency (n=27); group B with a risk of malnutrition (n=59); group C with malnutrition (n=29) | In group A (nutritional sufficiency): 24 (88.9%); in group B (malnutrition): 50 (84.7%); group C (suggestive of malnutrition): 27 (93.1%) | Median age: 66 (32-86) | Lung cancer | 100% | NR |
| Hwang *et al*. (2011) | Korea | Oncology- university hospital | To evaluate the relationships between carcinomatosis peritonei, liver metastasis, bone metastasis, ECOG PS, albumin, CRP, GPS, and PFS, and OS in patients with recurrent or metastatic gastric cancer receiving first-line palliative chemotherapy. | Total participants with cancer (n=402) | 293 (72.9%) | 203 (50.5%) of patients were < 60 years of age; 199 (49.5%) were ≥ 60 | Gastric adenocarcinoma | 77.6% | NR |
| Kwak *et al*. (2011) | Korea | Four hospice-palliative care centres | To examine fatigue and serum levels of IL-6 and TNF-α in terminally ill Korean cancer patients without clinical evidence of acute inflammation in order to clarify the roles of inflammatory cytokines in fatigue. | Total participants with advanced cancer (n=90); no control | 48 (53%) | 64.3 ± 12.7 (range NR) | Mixed | 100% | NR |
| Lee *et al*. (2011)b | Korea | Emergency | To investigate the relationship between serum CRP levels and the short-term mortality of advanced cancer in ED patients. | Total participants with advanced cancer (n=126) | 92 (73%) | 65.1 ± 11.3 (range NR) | Mixed | 100% | NR |
| Scheede-Bergdahl *et al*. (2011) | Canada | Nutrition and Performance Laboratory | To investigate the clinical relevance of plasma levels of four pro-inflammatory cytokines in advanced cancer patients to further establish their potential in the diagnostic definition of cancer cachexia. | Total participants with advanced cancer (n=83) | 47 (56.6%) | 61.8 ± 12.9 (34-85) | GI or NSCLC | 100%: 41% locally advanced and 59.0% metastatic | NR |
| Vlachostergios *et al*. (2011) | Greece | University hospital- oncology | To investigate the potential correlations of IGF-I with known clinical and biochemical predictors of adverse clinical outcome, including inflammatory response and weight loss, and examined their clinical relevance with regard to TTP and OS in patients with metastatic NSCLC. | Total participants with advanced cancer (n=77) | 66 (85.7%) | 49 (63.6%) of patients were ≤ 70 years old; 28 (36.4%) were > 70 years old | NSCLC | 100% | NR |
| Diakowska *et al*. (2010) | Poland | NR | To investigate the differences in serum leptin concentrations adjusted to gender and body mass in all these conditions as compared to healthy participants with reference to the severity of background inflammatory response. | Total participants (n=218); with cancer and cachexia (n=84); with cancer and no cachexia (n=51); with non-malignant cancer and cachexia (n=20); non-malignant cancer and non-cachectic (n=63) | In cancer cachexia group: 65 (77.3%); in non-cachexic cancer patients: 43 (84.3%); in non-malignant cachexia controls: 7 (23.3%) and in non-malignant non-cachectic group: 37 58.7%). | In cancer cachexia group: 63.3 (35-86); in non-cachexic cancer patients: 63.7 (24-83); in non-malignant cachexia controls: 65.2 (51-84) and in non-malignant non-cachectic group: 60.5 (47-82) | Esophageal cancer | 84% | 3 (2.2%) stage I, 18 (13.3%) stage II, 33 (24.4%) stage III and 81 (60%) stage IV |
| Meek *et al*. (2010) | UK | Oncology- hospital | To examine the relationship between IGF-1, IGFBP-3, weight loss and the systemic inflammatory response in patients with inoperable NSCLC. | Total participants with advanced cancer (n=56) | 34 (60.7%) | 11 patients <60 years old and 45 patients ≥ 60 years old. | NSCLC | 100% | 51.7% stage III, 46.4% stage IV |
| Ishizuka *et al*. (2009) | Japan | University hospital-Gastroenterological surgery | To evaluate the influence of the mGPS for prediction of mortality in these patients. | Total participants with advanced cancer (n=112) | 67 (59.8%) | 74 participants were ≤ 70 years old; and 38 > 70 years old | Colorectal Cancer | 100% | 2.7% stage IIB, 1.7% stage III and 95.6% stage IV |
| Karapanagiotou *et al*. (2009) | Greece | NR | To detect the role of ghrelin in cachexia and systemic inflammation of advanced NSCLC patients as well as its role as a diagnostic and prognostic tool. | Total participants (n=161); NSCLC patients with weight loss (n=75); NSCLC patients without weight loss (n=26); healthy controls (n=60) | In weight-loss group: 21 (84%); in the non-weight loss group: 62 (81.6%) | In the cachectic cancer group mean age: 59.9 ± 11.8; in the non-cachectic cancer group: 55.9 ± 10.7; in the control group: 52.1 ± 12.3 | NSCLC | 100% | 23 (23%) stage IIIB and 78 (77%) stage IV |
| Paddison *et al*. (2009) | USA | Palliative care | Primary aim: To investigate whether routinely collected cellular immune data were associated with the severity of fatigue reported by advanced lung cancer patients. Secondary aim: To examine whether the severity of fatigue was related to survival. | Total participants with advanced cancer (n=44) | 18 (40.9%) | 66 ± 8.3 (range NR) | NSCLC | 100% | All either Stage IIIB with effusion or Stage IV (stage % NR) |
| Takahashi *et al*. (2009) | Japan | Medical university | To examine plasma cytokine and hormone levels prospectively in cachectic cancer patients and healthy volunteers. | Total participants (n=26); cachectic cancer patients (n=16); healthy hospital personnel who had undergone no changes in body weight over the previous 6 months, had no acute or chronic disease, and were receiving no regular medication.(n=10) | 12 (75%) | 63 ± 11 (range NR) | Mixed | 100% | 100% stage IV |
| Inagaki *et al*. (2008) | Japan | NR | To investigate associations between plasma IL-6 levels and fatigue in terminally ill cancer patients. | Total participants with advanced cancer (n=46); clinically fatigued patients (n=27); without fatigue (n=19) | 28 (60.8%) | 58.4 ± 10.5 (range NR) | Mixed | 100% | NR |
| Karapanagiotou *et al*. (2008) | Greece | NR | To examine the diagnostic and prognostic role of leptin, adiponectin and resistin in advanced NSCLC, their association with cancer-related weight loss and the potential effect of chemotherapy on their serum levels. | Total participants (n=152); with advanced cancer (n=101); healthy controls (n=51) | In the cancer group: 83 (82%); in the control group: 26 (51%) | In the cancer group: 64.2 ± 10.4; in the healthy controls: 55.5 ± 8.9 | NSCLC | 100% | 23 (23%) stage IIIB and 78 (77%) stage IV |
| Sharma *et al*. (2008) | Australia | General hospital- unspecified | 1) To confirm the prognostic value of the GPS in advanced colorectal cancer, and 2) to explore a predictive pattern of plasma cytokines and their gene polymorphisms for clinical outcome; and 3) to investigate which cytokines contribute to GPS. | Total participants with advanced cancer (n=52) | 33 (64%) | 11 (21%) of patients were ≤ 60; 41 (79%) were >60 years old | Colorectal cancer | 100% | 100% stage IV |
| Weryńska *et al*. (2008) | Poland | NR | To evaluate serum leptin concentrations in the groups of lung cancer patients with and without cachexia when compared to healthy controls, and to explore the correlations between serum leptin concentration level and the antropometric indicators of cancer cachexia: body mass, arm circumference and skin triceps fold thickness. | Total participants with advanced cancer (n=40); with cachexia (n=20); no cachexia (n=20) | In the cancer group: 25 (62.5%); in the control group: 5 (33.3%) | Mean age in the cancer group: 61 (50–75); mean age in the control group: 44 (28-77) | NSCLC | 100% | 15% stage IIIA, 30% stage IIIB, 35% stage IV |
| Demiray *et al*. (2007) | Turkey | Oncology | To investigate the role of serum leptin and resistin levels in the pathogenesis of cancer cachexia to evaluate whether these peptides are effective in predicting cachexia and to investigate their effects on the quality of life of the patients. | Total participants (n=87); with advanced cancer (n=67); healthy individuals without a known chronic disease (n=20) | In the cancer group: 62 (92.5%); in the control group: 16 (80%). | Mean age in the cancer group: 62.9 ± 8.7; in the control group: 63.1 ± 6.2 (range NR) | NSCLC | 100% | Stage IIIB and stage IV (doesn't specify % of stage) |
| Ravasco *et al*. (2007) | Portugal | NR | To investigate the influence of inflammatory cytokines, pro-cachectic, immunomodulatory, and pro-angiogenic on REE, weight, and nutritional intake and to explore potential interactions between their circulating concentrations and colorectal cancer stage/histologic differentiation and response to radiotherapy. | Total participants with cancer (n=101) | 80 (79.2%) | 65 ± 12 (37-88) | Colorectal adenocarcinoma | 85% | 6.9% stage I, 7.9% stage II, 50.4% stage III, 34.6% stage IV |
| Richey *et al*. (2007) | USA | Patients were recruited from Head and Neck Tumor Board conferences and outpatient clinics | Primary objective: To more completely characterize cancer cachexia in HNSCC in terms of associated clinical variables, serum cytokines, measures of inflammation and anaemia, and cachexia factors. Secondary objective: To investigate tumour cytokine and cachexia factor expression. | Total participants with cancer (n=24); cachectic patients (n=11); non-cachectic patients (n=13) | In the cachectic group: 8 (73%); in the non-cachectic group: 2 (15%) | Mean age in the cachectic group: 57 ± 12; mean age in the non-cachectic group: 58 ± 9 (range NR) | HNSCC | 70.8% | 70.8% stage IV- other stages NR |
| Suh *et al*. (2007) | Korea | Palliative care | To prove serum CRP level as a predictor of survival time, considering patient’s symptoms, physical examination findings, and various serological variables in terminally ill cancer patients with a prospective cohort design. | Total participants with advanced cancer (n=44) | 25 (56.8%) | Median age: 68 years (30–87) | Mixed | 100% | NR |
| Al Murri *et al*. (2006) | UK | Oncology centre | To examine the relationship between the GPS and survival in patients with metastatic breast cancer. | Total participants with metastatic breast cancer (n=96) | All female (0%) | 21 (21.8%) patients were ≤ 50 years old; 75 (78.1%) were >50 years of age (range NR) | Breast | 100% | NR |
| Kayacan *et al*. (2006) | Turkey | NR | To determine the role TNF-α and IL-6, implicated for cancer cachexia development in inoperable NSCLC patients. | Total participants (n=56); with cancer (n=44; 23 cachectic and 21 non-cachectic); healthy smokers for the control (n=12) | 51 (91%) | In the cachexia group: 59.9 ± 11.8; in the non-cachectic group: 55.9 ± 10.7; in the control group: 52.1 ± 12.3 | NSCLC | 100% | In the cachexia group: 18 (60.9%) stage IV and 4 (17.4%) stage IIIb.  In the non-cachexia group: 10 (47.6%) stage IV and 6 (28.6%) stage IIIb. |
| Ramsey *et al*. (2006) | UK | Specialist renal cancer unit | To examine the value of the GPS, compared with established scoring systems, for predicting cancer-specific survival in patients with metastatic renal cancer | Total participants with advanced cancer (n=119) | 85 (70.8%) | 56 (47%) of patients were ≤ 60 years of age; 63 (52.9%) were > 60 years old | Renal cancer | 100% | NR |
| Di Nisio *et al*. (2005) | The Netherlands | NR | To evaluate: 1) the prognostic value for survival of circulating levels of IL-6, IL-10, IFN-a, and P-selectin in all the 141 patients at the time of entry into the study; 2) the association between these circulating markers and prognosis in the group of patients treated with LMWH; and 3) whether the beneficial survival effects observed in the MALT study were related to the influence of LMWH on plasma levels of soluble P-selectin or cytokines. | Total participants with advanced cancer (n=141) | 83 (58.8%) | 62.3 (38.4-85.7) | Mixed | 100% | NR |
| Rich *et al*. (2005) | France | General hospital- unspecified | To evaluate the role of circulating cytokines in the production of symptoms in cancer patients. | Total participants with advanced cancer (n=80); with near normal circadian rhythm (n=40); with dampened circadian rhythm (n=40) | In group 1 (good rhythm): 23 (57.5%); group 2 (dampened rhythm): 29 (72.5%) | Median age in group 1 (good rhythm): 59.5 (42-76); median age in group 2 (dampened rhythm): 60 (36-74) | Colorectal cancer | 100% | NR |
| Bolukbas *et al*. (2004) | Turkey | Hospital- oncology department | Primary aim: to evaluate the serum leptin concentration in patients with advanced gastrointestinal cancer and to determine the factors such as gender, age and BMI which may be related with this peptide in this participants. Secondary aim: to find out the relationship of leptin with weight loss and to compare the serum leptin concentrations in distinct type of gastrointestinal cancers. | Total participants (n=69); with advanced gastrointestinal cancer (n=44); healthy controls with stable weight (n=25) | In the cancer group: 29 (66%); in the non-cancer group: 12 (48%) | Median age in the gastric cancer group: 58 (range 34-80); in the colorectal cancer group: 59 (range 33-80); in the malignant group: 58 (range 33-80); and in the control group: 38 (range 22-67) | Gastrointestinal | 100% | 100% stage III |
| De Vita *et al*. (2004) | Italy | NR | To evaluate IL-6 serum levels and their prognostic significance in patients with advanced GI cancer. | Total participants with advanced cancer (n=68) | 46 (67.6%) | 34 (50%) of patients were ≤ 60 years of age; 34 (50%) were > 60 years old | Gastric (n=30) and colorectal (n=38) | 100% | 10.2% stage III, 89.7% stage IV |
| Dulger *et al*. (2004) | The Netherlands | NR | To investigate the serum levels of leptin, TNF-α , IL-1b, IL-6, insulin, and growth hormone in patients with upper Gl cancer and cachexia. | Total participants (n=54); with cancer cachexia (n=19); with cancer and no cachexia (n=20); healthy controls (n=15) | 25 (64%) | Median age: 53.72 (28-76) | Esophogeal | 100% | All stage IV |
| Elahi *et al*. (2004) | UK | Hospital- department of Clinical Biochemistry | To examine the relationship between the combination of hypoalbuminemia and an elevated circulating concentration of CRP and survival in patients with advanced GI cancer. | Total participants with advanced cancer (n=165) | 105 (64%) | 110 (67%) of patients were < 70 years old and 55 (33%) of patients were > 70 years old. | Gastric: 66 (40%) and colorectal: 99 (60%) cancer | 100% | NR |
| Jamieson *et al*. (2004) | UK | Palliative care | To examine the relationship between adiponectin and the systemic inflammatory response in weight-losing patients with NSCLC. | Total participants (n=33); with advanced cancer (n=20); healthy controls (n=13) | In the cancer group: 12 (65%); in the control group: 6 (46%) | Median age in the cancer group: 64 (43-79); in the control group: 65 (46-74) | NSCLC | 100% | NR |
| Songur *et al*. (2004) | Turkey | NR | To initiate a prospective clinical protocol for investigation of serum levels of IL-6 in advanced NSCLC patients and analyzed the influence on malnutrition and survival. | Total participants (n=91); with advanced cancer (n=71); healthy controls (n=20) | 65 (91.5%) | 38 patients < 60 years old; 33 patients ≥ 60 years old | NSCLC | 100% | 48% stage III, 52% stage IV |
| Scott *et al*. (2003) | UK | NR | To examine the relationships between weight loss, the systemic inflammatory response and quality of life in patients with inoperable NSCLC. | Total participants with advanced cancer (n=106); weight-loss group (n=45); weight-stable group (n=61) | 62 (58.4%) | Median age: 69 (43-87) | NSCLC | 100% | 73.6% stage III, 26.4% stage IV |
| Aleman *et al*. (2002) | Spain | NR | To analyse the relation of serum leptin levels with the nutritional status and the inflammatory response in patients with advanced NSCLC. | Total participants (n=106); with advanced cancer (n=76); without cancer (n=30) | 67 (88%) | Median age: 62.5 years (36–75) | NSCLC | 100% | 7.8% stage IIIA, 39.4% stage IIIB, 52.6% stage IV |
| Orditura *et al*. (2002) | Italy | NR | To determine if IL-8 serum levels may have prognostic significance in patients with advanced NSCLC. | Total participants (n=85); with advanced cancer (n=60); healthy controls (n=25) | 49 (81.6%) | 28 patients were ≤ 60 years old, and 32 patients > 60 years | NSCLC | 100% | 46.7% stage III, 53.3% stage IV |
| Scott *et al*. (2002) | UK | NR | To examine the relationship between the magnitude of the systemic inflammatory response and weight loss, PS and survival in patients with inoperable NSCLC. | Total participants with cancer (n=106) | 62 (58.4%) | Median age: 69 (43-87) | NSCLC | 100% | 73.6% stage III, 26.4% stage IV |
| Jatoi *et al*. (2001) | USA | NR | 1) To investigate whether circulating concentrations of NPY and leptin differ among cancer patients with advanced disease compared with normative values derived from a healthy control population, and 2) To explore whether serum concentrations of NPY, leptin, and/or CCK8 may be able to serve as correlates of anorexia severity in patients with advanced cancer. | NI | 48 (66%) | 62 (range 42-84) | NR | 100% | NR |
| Mantovani *et al*. (2001) | Italy | NR | To examine the correlation between serum levels of leptin, IL-6 and TNF-α in a population of non-cachectic but advanced-stage cancer patients at various sites and to determine the correlation between leptin and pro-inflammatory cytokines and the most relevant clinical parameters of patients, such as BMI and PS. | Total participants (n=58); with advanced cancer (n=29); healthy controls (n=29); | In the cancer group: 14 (48.2%); in the control group: 13 (44.8%) | Mean age in the cancer group: 55 (41-77); in the control group: 45 (20-80) | Mixed | 100% | 1 (3.4%) stage IIIA, 28 (96.6%) stage IV |
| Mantovani *et al*. (2000) | Italy | NR | To determine whether there is a relationship between the production and/or release of pro-inflammatory cytokines and leptin at the source cell level. | Total participants (n=32); with advanced cancer (n=16); healthy controls (n=16) | 8 (50%) | 58.3 (range 41–71) | Mixed | 100% | 10 patients (62.5%) with stage IV, and 6 (37.5% with stage III |
| Nenova *et al*. (2000) | Bulgaria | NR | To investigate the serum levels of TNF-α cytokine in advanced carcinoma patients and to attempt an evaluation of its prognostic significance and its relation to cancer cachexia. | Total participants (n=87); with advanced cancer (n=71); clinically healthy controls (n=16) | 20 (28.1%) | Average age 53.6 ± 1.8 years | Mixed | 100% | 100% stage IV |
| O'Gorman *et al*. (1999) | UK | NR | To examine the temporal relationship between weight loss, appetite, performance status, and acute-phase protein response in patients with GI cancer. | Total participants with cancer (n=50); with weight loss after 6-8 weeks of observation (n=16); with weight gain after 6-8 weeks (n=9); and patients who were stable after 6-8 weeks (n=25) | 35 (70%) | Median age: 68 (44-78) | Mixed | 100% | NR |
| Okada *et al*. (1998) | Japan | NR | To investigate the relationship between serum IL-6 levels and the clinical status of pancreatic cancer. | Total participants (n=100); with pancreatic cancer (n=55); patients with chronic pancreatitis (n=25); normal healthy adults (n=20) | 38 (69%) | 61.2 ± 7.3 (range NR) | Pancreatic cancer | 91% | 9.9% stage II, 30.1% stage II, 60% stage IV |
| Wallace *et al*. (1998) | UK | NR | NR | Total participants (n=54); with advanced cancer (n=27); healthy controls (n=27) | In the cancer group: 14 (82.3%); in the control group: 14 (51.8%) | Median age in the cancer group: 62 (range 48-74); median age in the control group: 59 (range 49-67) | Gastrointestinal | 100% | NR |
| Maltoni *et al*. (1997) | Italy | Palliative care centres | To better define the prognosis of terminal patients by evaluating the prognostic capacity of certain easily detectable biological parameters. | Total participants with advanced cancer (n=530) | 300 (57.8%) | 226 (43.5%) of patients were ≤ 65 years old; 293 (56.5%) were > 65 years old. | Mixed | 100% | NR |
| Simons *et al*. (1997) | The Netherlands | NR | To investigate the relationship between total plasma leptin, weight loss, body composition, appetite and REE in a group of male lung-cancer patients. | Total participants with cancer and weight loss of 10% pre-illness (n=21) | All male (100%) | Median age: 69 (56-82) | Lung | 76% | 23.8 stage III, 52.3% stage IV |

**Abbreviations:** ADL: Activities of daily living; BMI: Body Mass Index; CCS: Cancer-specific survival; CRF: Cancer-related fatigue; CRP: C-reactive protein; ECOG: The Eastern Cooperative Oncology Group; ED: Emergency department; EOC: Endothelial ovarian cancer; ESAS: Edmonton Symptom Assessment System; GI: Gastrointestinal; GPS: Glasgow Prognostic Score; IFN: Interferon; IGF: Insulin-like growth factor; IGFBP: Insulin-like growth factor binding protein; IL-: Interleukin; LMWH: Low molecular weight heparin; mGPS: Modified Glasgow Prognostic Score; MNA: Mini nutritional assessment; NLR: Neutrophil/Lymphocyte ratio; NPY: Neuropeptide Y; NR: Not reported; NSCLC: Non-small-cell lung carcinoma; OS: Overall survival; PFS: Progression free survival; PI: Prognostic index; PLR: Platelet/Lymphocyte ratio; PNI: Prognostic nutritional index; PROM: Patient-reported outcome measures; PS: Performance status; QOL: Quality of life; TNF: Tumor necrosis factor

1. Secondary analysis of Amano, 2016 [↑](#footnote-ref-1)
