# Supplementary material for: A systematic review of the overlap of fluid biomarkers in delirium and advanced cancer-related syndromes
Source: BMC Psychiatry. 2020 Apr 22;20:182. doi: 10.1186/s12888-020-02584-2 (PMC7178636; doi:10.1186/s12888-020-02584-2)
Supplement: Supplementary file 4 — Additional file 4:. Quality assessment of included delirium studies using the REMARK checklist The quality assessment for all included delirium studies. [file 12888_2020_2584_MOESM4_ESM.docx]

## Additional file 4: Quality assessment of included delirium studies using the REMARK checklist

| **Author(s), year** | **Population^1^** | **Assay** | | | | | | | | **Clinical endpoints^10^** | **Sample size calculation ^11^** | **Analysis** | |
| --- | --- | --- | --- | --- | --- | --- | --- | --- | --- | --- | --- | --- | --- |
|  |  | **Biological material^2^** | **Preservation/storage^3^** | **Assay method^4^** | **Reagents/kits^5^** | **Repeatability^6^** | **Time point^7^** | **Scoring of biomarker^8^** | **Blinding^9^** |  |  | **Statistical analysis ^12^** | **Covariates^13^** |
| Egberts *et al.* (2017) |  |  |  |  |  |  |  |  |  |  |  |  |  |
| Kozak *et al.* (2017) |  |  |  |  |  |  |  |  |  |  |  |  |  |
| Tomasi *et al.* (2017) |  |  |  |  |  |  |  |  |  |  |  |  |  |
| Vasunilashorn *et al.* (2017) |  |  |  |  |  |  |  |  |  |  |  |  |  |
| Chu *et al.* (2016) |  |  |  |  |  |  |  |  |  |  |  |  |  |
| Dillon *et al.* (2016) |  |  |  |  |  |  |  |  |  |  |  |  |  |
| Guo *et al.* (2016) |  |  |  |  |  |  |  |  |  |  |  |  |  |
| Karlicic *et al.* (2016) |  |  |  |  |  |  |  |  |  |  |  |  |  |
| Neerland *et al.* (2016) |  |  |  |  |  |  |  |  |  |  |  |  |  |
| Shen *et al.* (2016) |  |  |  |  |  |  |  |  |  |  |  |  |  |
| Sun *et al.*  (2016) |  |  |  |  |  |  |  |  |  |  |  |  |  |
| Yen *et al.*  (2016) |  |  |  |  |  |  |  |  |  |  |  |  |  |
| Avila-Funes *et al.*  (2015) |  |  |  |  |  |  |  |  |  |  |  |  |  |
| Brum *et al.* (2015) |  |  |  |  |  |  |  |  |  |  |  |  |  |
| Egberts *et al.* (2015) |  |  |  |  |  |  |  |  |  |  |  |  |  |
| Foroughan *et al.*  (2015) |  |  |  |  |  |  |  |  |  |  |  |  |  |
| Skrede *et al.*  (2015) |  |  |  |  |  |  |  |  |  |  |  |  |  |
| Vasunilashorn *et al.*  (2015) |  |  |  |  |  |  |  |  |  |  |  |  |  |
| Alexander *et al.*  (2014) |  |  |  |  |  |  |  |  |  |  |  |  |  |
| Baranyi *et al.*  (2014) |  |  |  |  |  |  |  |  |  |  |  |  |  |
| Cape *et al.*  (2014) |  |  |  |  |  |  |  |  |  |  |  |  |  |
| Capri *et al.*  (2014) |  |  |  |  |  |  |  |  |  |  |  |  |  |
| Chen *et al.* (2014) |  |  |  |  |  |  |  |  |  |  |  |  |  |
| Hatta *et al.*  (2014) |  |  |  |  |  |  |  |  |  |  |  |  |  |
| Kazmierski *et al.*  (2014) |  |  |  |  |  |  |  |  |  |  |  |  |  |
| Ritchie *et al.* (2014) |  |  |  |  |  |  |  |  |  |  |  |  |  |
| Ritter *et al.*  (2014) |  |  |  |  |  |  |  |  |  |  |  |  |  |
| Zhang *et al.* (2014) |  |  |  |  |  |  |  |  |  |  |  |  |  |
| Cerejeira *et al.*  (2013) |  |  |  |  |  |  |  |  |  |  |  |  |  |
| Colkesen *et al.*  (2013) |  |  |  |  |  |  |  |  |  |  |  |  |  |
| Kazmierski *et al.* (2013) |  |  |  |  |  |  |  |  |  |  |  |  |  |
| Kazmierski *et al.*  (2013)b |  |  |  |  |  |  |  |  |  |  |  |  |  |
| Liu *et al.* (2013) |  |  |  |  |  |  |  |  |  |  |  |  |  |
| Plaschke *et al.* (2013) |  |  |  |  |  |  |  |  |  |  |  |  |  |
| Skrobik *et al.* (2013) |  |  |  |  |  |  |  |  |  |  |  |  |  |
| Westhoff *et al.* (2013) |  |  |  |  |  |  |  |  |  |  |  |  |  |
| Bakker *et al.*  (2012) |  |  |  |  |  |  |  |  |  |  |  |  |  |
| Baranyi *et al.*  (2012) |  |  |  |  |  |  |  |  |  |  |  |  |  |
| Cerejeira *et al.*  (2012) |  |  |  |  |  |  |  |  |  |  |  |  |  |
| Girard *et al.*  (2012) |  |  |  |  |  |  |  |  |  |  |  |  |  |
| Osse *et al.*  (2012) |  |  |  |  |  |  |  |  |  |  |  |  |  |
| Bisschop *et al.*  (2011) |  |  |  |  |  |  |  |  |  |  |  |  |  |
| Holmes *et al.* (2011) |  |  |  |  |  |  |  |  |  |  |  |  |  |
| Lee *et al.*  (2011) |  |  |  |  |  |  |  |  |  |  |  |  |  |
| McGrane *et al.* (2011) |  |  |  |  |  |  |  |  |  |  |  |  |  |
| Morandi *et al.*  (2011) |  |  |  |  |  |  |  |  |  |  |  |  |  |
| Van der Boogaard *et al.* (2011)a |  |  |  |  |  |  |  |  |  |  |  |  |  |
| Van der Boogaard *et al.*  (2011)b |  |  |  |  |  |  |  |  |  |  |  |  |  |
| Burkhart *et al.*  (2010) |  |  |  |  |  |  |  |  |  |  |  |  |  |
| Mu *et al.*  (2010) |  |  |  |  |  |  |  |  |  |  |  |  |  |
| Pearson *et al.* (2010) |  |  |  |  |  |  |  |  |  |  |  |  |  |
| Plaschke *et al.* (2010) |  |  |  |  |  |  |  |  |  |  |  |  |  |
| Tsruta *et al.* (2010) |  |  |  |  |  |  |  |  |  |  |  |  |  |
| Van Munster *et al.* (2010) |  |  |  |  |  |  |  |  |  |  |  |  |  |
| Adamis *et al.* (2009) |  |  |  |  |  |  |  |  |  |  |  |  |  |
| Van Munster *et al.* (2009) |  |  |  |  |  |  |  |  |  |  |  |  |  |
| Lemstra *et al.* (2008) |  |  |  |  |  |  |  |  |  |  |  |  |  |
| Pfister *et al.* (2008) |  |  |  |  |  |  |  |  |  |  |  |  |  |
| Rudolph *et al.* (2008) |  |  |  |  |  |  |  |  |  |  |  |  |  |
| Van Munster *et al.* (2008) |  |  |  |  |  |  |  |  |  |  |  |  |  |
| Adamis *et al.* (2007) |  |  |  |  |  |  |  |  |  |  |  |  |  |
| de Rooij *et al.*  (2007) |  |  |  |  |  |  |  |  |  |  |  |  |  |
| Plaschke *et al.* (2007) |  |  |  |  |  |  |  |  |  |  |  |  |  |
| White *et al.* (2005) |  |  |  |  |  |  |  |  |  |  |  |  |  |
| Wilson *et al.* (2005) |  |  |  |  |  |  |  |  |  |  |  |  |  |
| Beloosesky *et al.* (2004) |  |  |  |  |  |  |  |  |  |  |  |  |  |
| Robertsson *et al.* (2001) |  |  |  |  |  |  |  |  |  |  |  |  |  |
| Van der Mast  *et al.* (2000) |  |  |  |  |  |  |  |  |  |  |  |  |  |
| Van der Mast *et al.*  (1999) |  |  |  |  |  |  |  |  |  |  |  |  |  |
| Gustafson *et al.*  (1993) |  |  |  |  |  |  |  |  |  |  |  |  |  |
| McIntosh *et al.*  (1985) |  |  |  |  |  |  |  |  |  |  |  |  |  |

| KEY | Yes | No | Unclear | N/A |
| --- | --- | --- | --- | --- |

^1^ Describe the characteristics (for example, disease stage or co-morbidities) of the study patients, including their source and inclusion and exclusion criteria.
^2^ Describes the type of biological material used (including control samples)
^3^ Describes the methods of preservation and storage
^4^ Specifies the assay method used and provides (or references) a detailed protocol
^5^ Specifies the specific reagents or kits used
^6^ Reports any reproducibility assessments
^7^ The time point of the assay in relation to delirium
^8^ Provides a scoring and reporting protocol
^9^ Specifies whether and how assays were performed blinded to the study endpoint.
^10^ Precisely define all clinical endpoints examined.
^11^ Gives a rationale for sample size; if the study was designed to detect a specified effect size, the study gives the target power and effect size.
^12^ Describes univariate or multivariate analysis in detail including which model was used and what was compared
^13^ For multivariate analysis only: justifies the covariates used in the multivariate model
