# Supplementary material for: A systematic review of the overlap of fluid biomarkers in delirium and advanced cancer-related syndromes
Source: BMC Psychiatry. 2020 Apr 22;20:182. doi: 10.1186/s12888-020-02584-2 (PMC7178636; doi:10.1186/s12888-020-02584-2)
Supplement: Supplementary file 5 — Additional file 5:. Quality assessment of included cancer studies using the REMARK checklist The quality assessment for all included cancer studies. [file 12888_2020_2584_MOESM5_ESM.docx]

## Additional file 5: Quality assessment of included cancer studies using the REMARK checklist

| **Author(s), year** | **Population^1^** | **Assay** | | | | | | | | **Clinical endpoints^10^** | **Sample size calculation^11^** | **Analysis** | |
| --- | --- | --- | --- | --- | --- | --- | --- | --- | --- | --- | --- | --- | --- |
|  |  | **Biological material^2^** | **Preservation/storage^3^** | **Assay method^4^** | **Reagents/ kits^5^** | **Repeatability^6^** | **Time point^7^** | **Scoring of biomarkers^8^** | **Blinding^9^** |  |  | **Statistical analysis^12^** | **Covariates ^13^** |
| Amano *et al*. (2017) |  |  |  |  |  |  |  |  |  |  |  |  |  |
| Fogelman *et al*. (2017) |  |  |  |  |  |  |  |  |  |  |  |  |  |
| Luo *et al*. (2017) |  |  |  |  |  |  |  |  |  |  |  |  |  |
| Paulsen *et al*. (2017) |  |  |  |  |  |  |  |  |  |  |  |  |  |
| Amano *et al*. (2016) |  |  |  |  |  |  |  |  |  |  |  |  |  |
| Bye *et al*. (2016) |  |  |  |  |  |  |  |  |  |  |  |  |  |
| Mitsunga *et al*. (2016) |  |  |  |  |  |  |  |  |  |  |  |  |  |
| Morgado *et al*. (2016) |  |  |  |  |  |  |  |  |  |  |  |  |  |
| Rodrigues *et al*. (2016) |  |  |  |  |  |  |  |  |  |  |  |  |  |
| Srdic *et al*. (2016) |  |  |  |  |  |  |  |  |  |  |  |  |  |
| Wu *et al*. (2016) |  |  |  |  |  |  |  |  |  |  |  |  |  |
| Bilir *et al*. (2015) |  |  |  |  |  |  |  |  |  |  |  |  |  |
| Miura *et al*. (2015) |  |  |  |  |  |  |  |  |  |  |  |  |  |
| Miura *et al*. (2015)b |  |  |  |  |  |  |  |  |  |  |  |  |  |
| Barrera *et al*. (2014) |  |  |  |  |  |  |  |  |  |  |  |  |  |
| Blakely *et al*. (2014) |  |  |  |  |  |  |  |  |  |  |  |  |  |
| Fujiwara *et al*. (2014) |  |  |  |  |  |  |  |  |  |  |  |  |  |
| Lindemann *et al*. (2014) |  |  |  |  |  |  |  |  |  |  |  |  |  |
| Mondello *et al*. (2014) |  |  |  |  |  |  |  |  |  |  |  |  |  |
| Moriwaki *et al*. (2014) |  |  |  |  |  |  |  |  |  |  |  |  |  |
| Szkandera *et al*. (2014) |  |  |  |  |  |  |  |  |  |  |  |  |  |
| Zhang *et al*. (2014) |  |  |  |  |  |  |  |  |  |  |  |  |  |
| Jafri *et al*. (2013) |  |  |  |  |  |  |  |  |  |  |  |  |  |
| Laird *et al*. (2013) |  |  |  |  |  |  |  |  |  |  |  |  |  |
| Laird *et al*. (2013)b |  |  |  |  |  |  |  |  |  |  |  |  |  |
| Paiva *et al*. (2013) |  |  |  |  |  |  |  |  |  |  |  |  |  |
| Suh *et al*. (2013) |  |  |  |  |  |  |  |  |  |  |  |  |  |
| De Raaf *et al*. (2012) |  |  |  |  |  |  |  |  |  |  |  |  |  |
| Gioulbasanis *et al*. (2012) |  |  |  |  |  |  |  |  |  |  |  |  |  |
| Gulen *et al*. (2012) |  |  |  |  |  |  |  |  |  |  |  |  |  |
| Heitzer *et al*. (2012) |  |  |  |  |  |  |  |  |  |  |  |  |  |
| Minton *et al*. (2012) |  |  |  |  |  |  |  |  |  |  |  |  |  |
| Partridge *et al*. (2012) |  |  |  |  |  |  |  |  |  |  |  |  |  |
| Pond *et al*. (2012) |  |  |  |  |  |  |  |  |  |  |  |  |  |
| Wang *et al*. (2012) |  |  |  |  |  |  |  |  |  |  |  |  |  |
| Aydin *et al*. (2011) |  |  |  |  |  |  |  |  |  |  |  |  |  |
| Dev *et al*. (2011) |  |  |  |  |  |  |  |  |  |  |  |  |  |
| Gioulbasanis *et al*. (2011) |  |  |  |  |  |  |  |  |  |  |  |  |  |
| Hwang *et al*. (2011) |  |  |  |  |  |  |  |  |  |  |  |  |  |
| Kwak *et al*. (2011) |  |  |  |  |  |  |  |  |  |  |  |  |  |
| Lee *et al*. (2011)b |  |  |  |  |  |  |  |  |  |  |  |  |  |
| Scheede-Bergdahl *et al*. (2011) |  |  |  |  |  |  |  |  |  |  |  |  |  |
| Vlachostergios *et al*. (2011) |  |  |  |  |  |  |  |  |  |  |  |  |  |
| Diakowska *et al*. (2010) |  |  |  |  |  |  |  |  |  |  |  |  |  |
| Meek *et al*. (2010) |  |  |  |  |  |  |  |  |  |  |  |  |  |
| Ishizuka *et al*. (2009) |  |  |  |  |  |  |  |  |  |  |  |  |  |
| Karapanagiotou *et al*. (2009) |  |  |  |  |  |  |  |  |  |  |  |  |  |
| Paddison *et al*. (2009) |  |  |  |  |  |  |  |  |  |  |  |  |  |
| Takahashi *et al*. (2009) |  |  |  |  |  |  |  |  |  |  |  |  |  |
| Inagaki *et al*. (2008) |  |  |  |  |  |  |  |  |  |  |  |  |  |
| Karapanagiotou *et al*. (2008) |  |  |  |  |  |  |  |  |  |  |  |  |  |
| Sharma *et al*. (2008) |  |  |  |  |  |  |  |  |  |  |  |  |  |
| Weryńska *et al*. (2008) |  |  |  |  |  |  |  |  |  |  |  |  |  |
| Demiray *et al*. (2007) |  |  |  |  |  |  |  |  |  |  |  |  |  |
| Ravasco *et al*. (2007) |  |  |  |  |  |  |  |  |  |  |  |  |  |
| Richey *et al*. (2007) |  |  |  |  |  |  |  |  |  |  |  |  |  |
| Suh *et al*. (2007) |  |  |  |  |  |  |  |  |  |  |  |  |  |
| Al Murri *et al*. (2006) |  |  |  |  |  |  |  |  |  |  |  |  |  |
| Kayacan *et al*. (2006) |  |  |  |  |  |  |  |  |  |  |  |  |  |
| Ramsey *et al*. (2006) |  |  |  |  |  |  |  |  |  |  |  |  |  |
| Di Nisio *et al*. (2005) |  |  |  |  |  |  |  |  |  |  |  |  |  |
| Rich *et al*. (2005) |  |  |  |  |  |  |  |  |  |  |  |  |  |
| Bolukbas *et al*. (2004) |  |  |  |  |  |  |  |  |  |  |  |  |  |
| De Vita *et al*. (2004) |  |  |  |  |  |  |  |  |  |  |  |  |  |
| Dulger *et al*. (2004) |  |  |  |  |  |  |  |  |  |  |  |  |  |
| Elahi *et al*. (2004) |  |  |  |  |  |  |  |  |  |  |  |  |  |
| Jamieson *et al*. (2004) |  |  |  |  |  |  |  |  |  |  |  |  |  |
| Songur *et al*. (2004) |  |  |  |  |  |  |  |  |  |  |  |  |  |
| Scott *et al*. (2003) |  |  |  |  |  |  |  |  |  |  |  |  |  |
| Aleman *et al*. (2002) |  |  |  |  |  |  |  |  |  |  |  |  |  |
| Orditura *et al*. (2002) |  |  |  |  |  |  |  |  |  |  |  |  |  |
| Scott *et al*. (2002) |  |  |  |  |  |  |  |  |  |  |  |  |  |
| Jatoi *et al*. (2001) |  |  |  |  |  |  |  |  |  |  |  |  |  |
| Mantovani *et al*. (2001) |  |  |  |  |  |  |  |  |  |  |  |  |  |
| Mantovani *et al*. (2000) |  |  |  |  |  |  |  |  |  |  |  |  |  |
| Nenova *et al*. (2000) |  |  |  |  |  |  |  |  |  |  |  |  |  |
| O'Gorman *et al*. (1999) |  |  |  |  |  |  |  |  |  |  |  |  |  |
| Okada *et al*. (1998) |  |  |  |  |  |  |  |  |  |  |  |  |  |
| Wallace *et al*. (1998) |  |  |  |  |  |  |  |  |  |  |  |  |  |
| Maltoni *et al*. (1997) |  |  |  |  |  |  |  |  |  |  |  |  |  |
| Simons *et al*. (1997) |  |  |  |  |  |  |  |  |  |  |  |  |  |

| KEY | Yes | No | Unclear | N/A |
| --- | --- | --- | --- | --- |

^1^ Describe the characteristics (for example, disease stage or co-morbidities) of the study patients, including their source and inclusion and exclusion criteria.
^2^ Describes the type of biological material used (including control samples)
^3^ Describes the methods of preservation and storage
^4^ Specifies the assay method used and provides (or references) a detailed protocol
^5^ Specifies the specific reagents or kits used
^6^ Reports any reproducibility assessments
^7^ The time point of the assay in relation to the patients clinical course
^8^ Provides a scoring and reporting protocol
^9^ Specifies whether and how assays were performed blinded to the study endpoint
^10^ Precisely define all clinical endpoints examined.
^11^ Gives a rationale for sample size; if the study was designed to detect a specified effect size, the study gives the target power and effect size.
^12^ Describes univariate or multivariate analysis in detail including which model was used and what was compared
^13^ For multivariate analysis only: justifies the covariates used in the multivariate model
